# Novel sulfonamidospirobifluorenes as fluorescent sensors for mercury(ii) ion and glutathione†

**DOI:** 10.1039/c9ra00004f

**Published:** 2019-04-11

**Authors:** Komthep Silpcharu, Mongkol Sukwattanasinitt, Paitoon Rashatasakhon

**Affiliations:** Department of Chemistry, Faculty of Science, Chulalongkorn University Bangkok 10330 Thailand; Nanotec-CU Center of Excellence on Food and Agriculture, Department of Chemistry, Faculty of Science, Chulalongkorn University Bangkok 10330 Thailand paitoon.r@chula.ac.th +66 (2) 2187598 +66 (2) 2187633

## Abstract

Novel spirobifluorene derivatives containing two and four sulfonamide groups are successfully synthesized from the commercially available bromo-9,9′-spirobifluorene by Sonogashira couplings. These compounds exhibit an excellent selective fluorescence quenching by Hg(ii) in DMSO/HEPES buffer mixture with three-times-noise detection limits of 10.4 to 103.8 nM. A static aggregation induced quenching mechanism is proposed based on the data from ^1^H-NMR and UV-Vis spectroscopy, as well as the observation of the Tyndall effect. Quantifications of Hg(ii) using these sensors are in good agreement with those obtained from ICP-OES. The reversibility of these sensors is demonstrated by a complete fluorescence restoration upon addition of EDTA or l-glutathione. The application as a turn-on sensor for l-glutathione is demonstrated in a quantitative analysis of three samples of l-glutathione supplement drinks.

## Abstract 2

Two sulfonamide derivatives of spirobifluorene are synthesized and used as turn-off sensors for Hg(ii) ion. The signal restoration of the sensors by l-glutathione leads to a quantitative analysis of three supplement drinks.

## Graphical abstract

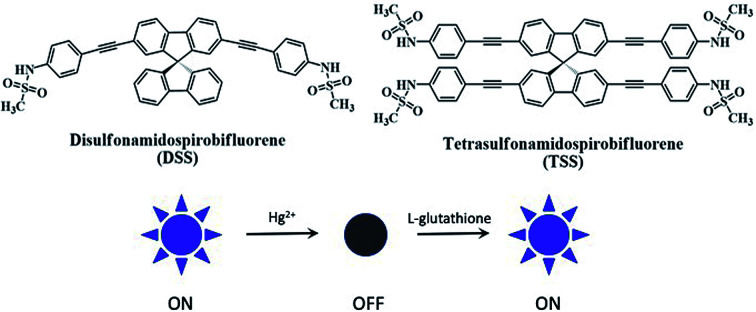

## 1. Introduction

Mercury is a highly toxic metal element. The World Health Organization (WHO)^1^ has recommended reducing and eliminating the use of mercury in the environment because it causes several diseases such as Minamata disease^2^ and the risk of heart disease.^3^ However, mercury and its derivatives are used in medicinal,^4^ agricultural,^5^ and many other industrial applications.^6^ Hence, close monitoring of mercury and its ionic species that contaminate the environment is of great significance. Several techniques such as atomic absorption spectroscopy (AAS),^7^ atomic emission spectroscopy (AES),^8^ inductively coupled plasma-mass spectrometry (ICP-MS),^9^ and inductively coupled plasma-optical emission spectrometry (ICP-OES)^10^ are routinely used for the analysis of mercury. Even though these methods can provide high sensitivity, accuracy, and precision, they are not practical for real-time or on-site analysis. In addition, they require well-trained specialists and high instrument maintenance costs, which can be avoided using selective fluorescent recognition probes.

Recent examples of selective fluorescent probes for Hg(ii) are the derivatives of triarylamine–triazine,^11^ rhodamine,^12^ bis-thiophene,^13^ and coumarin.^14^ During our research project on design and synthesis of selective fluorescent sensors, we became interested in spirobifluorene since it has high chemical and thermal stabilities with strong emission signal.^15^ Several derivatives of spirobifluorene are used as materials in optoelectronic devices such as field-effect transistors (FET),^16^ phototransistors,^17^ solar cells,^18^ and organic light-emitting diodes.^19^ There are a few examples of spirobifluorenes used as fluorescent chemosensors for analytes such as nitric oxide,^20^ H^+^,^21^ Ag(i),^22^ and Hg(ii) ion.^23^ The sensing mechanism for Hg(ii) is mostly involved specific hydrolysis reactions of thioacetals with Hg(ii).^24^ Although these sensors exhibited excellent selectivity towards Hg(ii), the irreversibility of hydrolysis reaction prevents further applications as a turn-on sensor for a secondary analyte.

**Fig. 1:**
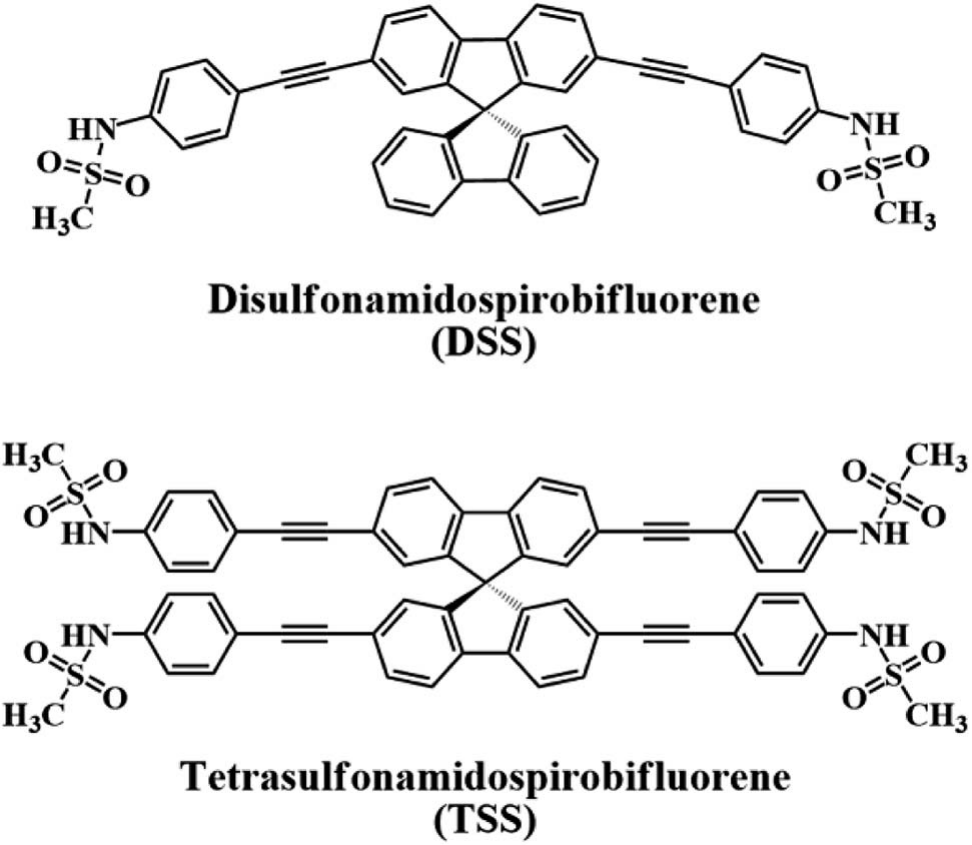
The structures of DSS and TSS.

In this work, we report our synthesis and application of two new derivatives of spirobifluorene named disulfonamidospirobifluorene (DSS) and tetrasulfonamidospirobifluorene (TSS) (Fig. 1). The sulfonamide groups are expected to function as specific binding sites for Hg(ii).^25^ This reversible complex formation may allow an application of this system as a turn-on fluorescent sensor for a secondary analyte such as cysteine and l-glutathione that can form a more stable complex with Hg(ii).^26^l-Glutathione is a value-added ingredient in a variety of health supplements as it helps reduce oxidative stress,^27^ and symptoms of neurodegenerative diseases such as Alzheimer,^28^ and Parkinson's disease.^29^ The quantitative analysis of l-glutathione in health supplements by a competitive binding with Hg(ii) in the DSS–Hg(ii) complex will also be demonstrated.

## 2. Experimental

### 2.1 Chemicals and materials

All reagents and solvents were purchased from Merck (Germany), Sigma-Aldrich (USA), or Fluka (Switzerland). Unless stated otherwise, these chemicals were used without further purification. All column chromatography were operated using silica gel 60 (70–230 mesh) purchased from Merck. Water used in all spectroscopic experiments was deionized with a Milli-Q® reference water purification system (Merck) to a specific resistivity of 18.2 MΩ cm. The stock solutions of DSS and TSS were prepared in dimethyl sulfoxide (DMSO) at 1 mM and diluted further to obtain expected concentrations. The stock solutions of metal ions in deionized water were prepared at 10 mM using the following commercially available salts: LiNO_3_, NaNO_3_, KNO_3_, AgNO_3_, Ca(NO_3_)_2_, Mg(NO_3_)_2_, Ba(NO_3_)_2_, Co(NO_3_)_2_, Cd(NO_3_)_2_, Zn(NO_3_)_2_, Pb(NO_3_)_2_, Ni(NO_3_)_2_, Cu(NO_3_)_2_, Hg(OAc)_2_, Fe(OAc)_2_, Fe(NO_3_)_3_, Al(NO_3_)_3_, Bi(NO_3_)_3_, Cr(NO_3_)_3_, and AuCl_3_. The pH-controlled and buffer solutions in deionized water (10 mM) were NaCl/HCl buffer (pH 1–2), acetate buffer (pH 3–5), HEPES buffer (pH 6–8), and glycine/NaOH buffer (pH 9–10).

### 2.2 Instruments

^1^H and ^13^C NMR spectra were collected on a 400 MHz NMR spectrometer which operated at 400 MHz for ^1^H and 100 MHz for ^13^C (Bruker Company). Mass spectra were recorded on a Microflex MALDI-TOF mass spectrometer (Bruker Daltonics). High-resolution mass spectra were obtained from a triple quadrupole GC/MS (Agilent Technologies). Elemental analysis was obtained from a CHNS/O analyzer (Flash 2000, Thermo Scientific). Absorption spectra were obtained from a UV-2250 UV-Vis Spectrophotometer (SHIMADZU, Japan) and emission spectra were obtained from a Carry Eclipse Fluorescence Spectrophotometer (Agilent Technologies). The absolute fluorescence quantum yields and lifetimes were determined using FLS980 Spectrometer (Edinburgh Instruments). The pH buffers were measured from an Ohaus pH meter. Amounts of Hg(ii) ion in water samples were verified by an inductively coupled plasma-optical emission spectrometer (ICP-OES) (iCAP 6500, Thermo Scientific).

### 2.3 Synthesis and characterizations

#### 2.3.1 Synthesis of 2,7-di(trimethylsilylethynyl)-9,9′-spirobifluorene (2a)

A mixture of 2,7-dibromo-9,9′-spirobifluorene (1a, 0.50 g, 1.05 mmol), Pd(PPh_3_)_2_Cl_2_ (74.0 mg, 0.11 mmol), CuI (40.0 mg, 0.21 mmol), PPh_3_ (55.6 mg, 0.21 mmol), and (*n*-Bu)_4_NBr (34.0 mg, 0.11 mmol) was dissolved in toluene (5 mL) and diisopropylamine (5 mL) under nitrogen atmosphere. After 15 minutes of stirring at ambient temperature, trimethylsilylacetylene (0.62 g, 6.33 mmol) was added dropwise, and the reaction was heated at 100 °C for 24 h. The crude was extracted with CH_2_Cl_2_ and 30% NH_4_Cl solution. The organic phase was washed deionized water and brine, dried over anhydrous Na_2_SO_4_, filtered, and concentrated under reduced pressure. The crude residue was purified by silica gel column chromatography with hexane as the eluent. A light-yellow solid of 2a was obtained in 0.51 g (95%). ^1^H-NMR (400 MHz, CDCl_3_) *δ* ppm 7.93 (d, *J* = 7.4 Hz, 1H), 7.83 (d, *J* = 7.8 Hz, 1H), 7.58 (d, *J* = 7.8 Hz, 1H), 7.45 (t, *J* = 7.3 Hz, 1H), 7.19 (t, *J* = 7.3 Hz, 1H), 6.95 (s, 1H), 6.79 (d, *J* = 7.4 Hz, 1H), 0.24 (s, 9H), ^13^C-NMR (100 MHz, CDCl_3_) *δ* ppm 149.2, 147.7, 141.9, 141.4, 132.1, 128.2, 128.1, 127.8, 124.3, 122.9, 120.28, 120.27, 105.4, 95.0, 65.7, 0.1. MS (MALDI-TOF) calcd. for C_35_H_32_Si_2_ ([M]^+^) 508.204, found 507.473.

#### 2.3.2 Synthesis of 2,2′,7,7′-tetra(trimethylsilylethynyl)-9,9′-spirobifluorene (2b)

A mixture of 2,2′,7,7′-tetrabromo-9,9′-spirobifluorene (1b, 0.50 g, 0.79 mmol), Pd(PPh_3_)_2_Cl_2_ (55.5 mg, 0.08 mmol), CuI (30.1 mg, 0.16 mmol), and PPh_3_ (41.5 mg, 0.16 mmol) was dissolved in toluene (5 mL) and diisopropylamine (5 mL) under nitrogen atmosphere. After 15 minutes of stirring at ambient temperature, trimethylsilylacetylene (0.47 g, 4.77 mmol) was added dropwise, and the reaction was heated at 100 °C for 24 h. The crude was extracted with CH_2_Cl_2_ and 30% NH_4_Cl solution. The organic phase was washed deionized water and brine, dried over anhydrous Na_2_SO_4_, filtered, and concentrated under reduced pressure. The crude residue was purified by silica gel column chromatography with hexane as the eluent. A white solid of 2b was obtained in 0.52 g (94%). ^1^H-NMR (400 MHz, CDCl_3_) *δ* ppm 7.77 (d, *J* = 7.9 Hz, 1H), 7.52 (d, *J* = 7.9 Hz, 1H), 6.81 (s, 1H), 0.18 (s, 9H), ^13^C-NMR (100 MHz, CDCl_3_) *δ* ppm 148.1, 141.4, 132.4, 127.8, 123.1, 120.4, 105.1, 95.3, 65.2, 0.0. MS (MALDI-TOF) calc. mass for C_45_H_48_Si_4_ ([M]^+^) 700.283, found 699.728.

#### 2.3.3 Synthesis of 2,7-diethynyl-9,9′-spirobifluorene (3a)

To a solution of 2a (0.50 g, 0.98 mmol) in CH_2_Cl_2_ (16 mL) was added a solution of NaOH (0.39 g, 9.83 mmol) in MeOH (4 mL) under nitrogen atmosphere. The reaction was stirred at room temperature for 15 h. The reaction mixture was extracted with CH_2_Cl_2_ and deionized water. The organic phase was washed brine, dried over anhydrous Na_2_SO_4_, filtered, and concentrated under reduced pressure. The crude mixture was purified by silica gel column chromatography with 5% CH_2_Cl_2_ in hexane as the eluent. A light-yellow solid of 3a was obtained in 0.36 g (100%). ^1^H-NMR (400 MHz, CDCl_3_) *δ* ppm 7.86 (d, *J* = 7.6 Hz, 1H), 7.80 (d, *J* = 7.9 Hz, 1H), 7.53 (d, *J* = 7.9 Hz, 1H), 7.40 (t, *J* = 7.5 Hz, 1H), 7.14 (t, *J* = 7.5 Hz, 1H), 6.89 (s, 1H), 6.73 (d, *J* = 7.6 Hz, 1H), 2.98 (s, 1H), ^13^C-NMR (100 MHz, CDCl_3_) *δ* ppm 149.4, 147.6, 141.9, 141.6, 132.2, 128.2, 128.1, 128.0, 124.2, 121.9, 120.4, 120.3, 83.9, 77.9, 65.7. MS (MALDI-TOF) calc. mass for C_29_H_16_ ([M]^+^) 364.125, found 363.221.

#### 2.3.4 Synthesis of 2,2′,7,7′-tetraethynyl-9,9′-spirobifluorene (3b)

To a solution of 2b (0.46 g, 0.65 mmol) in CH_2_Cl_2_ (16 mL) was added a solution of NaOH (0.26 g, 6.50 mmol) in MeOH (4 mL) under nitrogen atmosphere. The reaction was stirred at room temperature for 24 h. The reaction mixture was extracted with CH_2_Cl_2_ and deionized water. The organic phase was washed brine, dried over anhydrous Na_2_SO_4_, filtered, and concentrated under reduced pressure. The crude residue was purified by silica gel column chromatography with 5% CH_2_Cl_2_ in hexane as the eluent. A light-yellow solid of 3b was obtained in 0.27 g (100%). ^1^H-NMR (400 MHz, CDCl_3_) *δ* ppm 7.79 (d, *J* = 7.9 Hz, 1H), 7.54 (d, *J* = 7.9 Hz, 1H), 6.85 (s, 1H), 3.01 (s, 1H), ^13^C-NMR (100 MHz, CDCl_3_) *δ* ppm 148.0, 141.6, 132.6, 127.9, 122.2, 120.6, 83.7, 78.2, 65.2. MS (MALDI-TOF) calc. mass for C_33_H_16_ ([M]^+^) 412.125, found 411.361.

#### 2.3.5 Synthesis of *N*-(4-iodophenyl)methanesulfonamide (CH_3_SO_2_NH-C_6_H_4_-I)

4-Iodoaniline (2.00 g, 9.13 mmol) was dissolved in CH_2_Cl_2_ (5 mL) and pyridine (0.81 mL, 10.04 mmol) at 0 °C, and methanesulfonyl chloride (0.88 mL, 11.41 mmol) was slowly added dropwise. The reaction was allowed to warm at room temperature and stirred for 15 h. The reaction mixture was extracted with CH_2_Cl_2_ and 3N NaOH solution three times. The combined aqueous phase was acidified by concentrated HCl, and a white solid of CH_3_SO_2_NH-C_6_H_4_-I was filtered, washed with distilled water, and dried under vacuum. The product was obtained in 2.59 g (95%). ^1^H-NMR (400 MHz, acetone-D_6_) *δ* ppm 8.68 (s, 1H), 7.69 (d, *J* = 8.6 Hz, 2H), 7.16 (d, *J* = 8.6 Hz, 2H), 3.02 (s, 3H), ^13^C-NMR (100 MHz, acetone-D_6_) *δ* ppm 139.3, 139.1, 122.9, 87.9, 39.5. HRMS (ESI) calc. mass for C_7_H_7_INO_2_S^−^ ([M−H]^−^) 295.92423, found 295.94424.

#### 2.3.6 Synthesis of disulfonamidospirobifluorene (DSS)

A mixture of CH_3_SO_2_NH-C_6_H_4_-I (0.73 g, 2.47 mmol), Pd(PPh_3_)_4_ (95.1 mg, 0.08 mmol), CuI (15.6 mg, 0.08 mmol), and PPh_3_ (10.8 mg, 0.04 mmol) were dissolved in toluene (4 mL) and diisopropylamine (5 mL) under nitrogen atmosphere. After 15 minutes of stirring at room temperature, 3a (0.15 g, 0.41 mmol) that was dissolved in DMSO (1 mL) was dropped slowly *via* a syringe over a period of 5 minutes, and the reaction was heated at 50 °C for 24 h. The crude was extracted with CH_2_Cl_2_ and 30% w/v NH_4_Cl. The organic phase was washed deionized water and brine, dried over anhydrous Na_2_SO_4_, filtered, and concentrated under reduced pressure. The crude residue was purified by silica gel column chromatography with 60% ethyl acetate in hexane as the eluent. A light-brown solid of DSS was obtained in 0.20 g (69%). ^1^H-NMR (400 MHz, CDCl_3_) *δ* ppm 7.88 (d, *J* = 7.6 Hz, 1H), 7.82 (d, *J* = 7.9 Hz, 1H), 7.55 (d, *J* = 8.0 Hz, 1H), 7.41 (t, *J* = 7.5 Hz, 1H), 7.38 (d, *J* = 8.3 Hz, 2H), 7.33 (s, 1H), 7.16 (d, *J* = 8.0 Hz, 2H), 7.15 (t, *J* = 7.4 Hz, 1H), 6.92 (s, 1H), 6.77 (d, *J* = 7.5 Hz, 1H), 3.00 (s, 3H), ^13^C-NMR (100 MHz, CDCl_3_) *δ* ppm 149.4, 147.8, 141.9, 141.3, 136.8, 133.0, 131.7, 128.23, 128.17, 127.4, 124.3, 122.8, 120.4, 120.3, 120.03, 119.96, 90.2, 89.4, 65.8, 39.7. MS (MALDI-TOF) calc. mass for C_43_H_30_N_2_O_4_S_2_ ([M]^+^) 702.165, found 701.918. Elemental analysis (%) calc. for C_43_H_30_N_2_O_4_S_2_: C, 73.48; H, 4.30; N, 3.99. Found: C, 73.26; H, 4.48; N, 3.75.

#### 2.3.7 Synthesis of tetrasulfonamidospirobifluorene (TSS)

A mixture of CH_3_SO_2_NH-C_6_H_4_-I (0.86 g, 2.91 mmol), Pd(PPh_3_)_4_ (56.0 mg, 0.05 mmol), CuI (9.2 mg, 0.05 mmol), and PPh_3_ (12.7 mg, 0.05 mmol) were dissolved in toluene (4 mL), and diisopropylamine (4 mL) under nitrogen atmosphere. After 15 minutes of stirring at room temperature, 3b (0.10 g, 0.24 mmol) that was dissolved in DMSO (2 mL) was dropped slowly *via* a syringe over a period of 5 minutes. The reaction was heated at 50 °C for 48 h, allowed to cool to room temperature, and extracted with CH_2_Cl_2_ and 30% w/v NH_4_Cl. The organic phase was washed with deionized water and brine, dried over anhydrous Na_2_SO_4_, filtered, and concentrated under reduced pressure. The crude mixture was purified by silica gel column chromatography with 80% ethyl acetate in hexane as the eluent. A light-brown solid of TSS was obtained in 0.04 g (15%). ^1^H-NMR (400 MHz, acetone-D_6_) *δ* ppm 8.77 (s, 1H), 8.09 (d, *J* = 7.9 Hz, 1H), 7.60 (d, *J* = 7.9 Hz, 1H), 7.42 (d, *J* = 8.7 Hz, 2H), 7.28 (d, *J* = 8.7 Hz, 2H), 6.92 (s, 1H), 3.00 (s, 3H), ^13^C-NMR (100 MHz, acetone-D_6_) *δ* ppm 149.3, 142.2, 139.8, 133.5, 132.7, 127.5, 124.0, 122.1, 120.3, 119.2, 90.8, 89.8, 66.1, 39.8. MS (MALDI-TOF) calc. mass for C_61_H_44_N_4_O_8_S_4_ ([M]^+^) 1088.204, found 1089.066. Elemental analysis (%) calc. for C_61_H_44_N_4_O_8_S_4_: C, 67.26; H, 4.07; N, 5.14. Found: C, 67.01; H, 4.30; N, 4.99.

### 2.4 Quantum yield determinations

The excitation wavelengths for DSS and TSS were set at 360 nm and the integrated area of the emission signals was set from 370 to 600 nm. The signal rate on the instrument was set to 1 × 10^6^ counts per second. The emission signals were detected on the integrating sphere and analyzed by F980 software (Edinburgh instrument).

## 3. Results and discussion

### 3.1 Synthesis

**Scheme 1:**
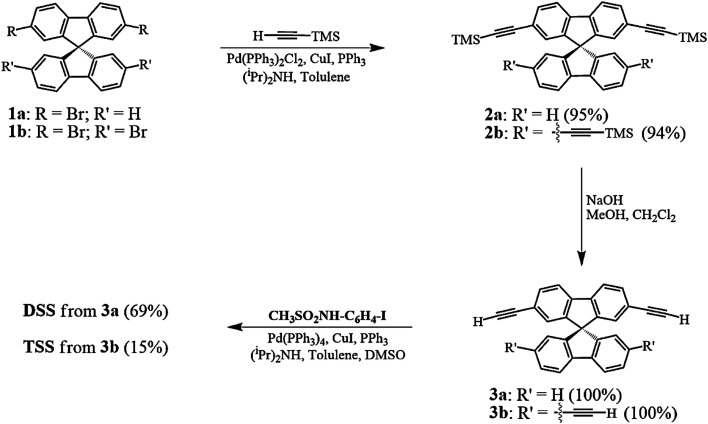
Synthesis of the DSS and TSS.

The synthesis of DSS and TSS (Scheme 1) began with a Sonogashira coupling between the commercially available brominated spirobifluorenes (1a, b). Both substrates were effectively coupled with trimethylsilylacetylene in excellent yields. After a treatment with NaOH in MeOH and CH_2_Cl_2_, the corresponding terminal alkyne 3a and 3b were produced in quantitative yields. In the last step, 3a and 3b were reacted with *N*-(4-iodophenyl)methanesulfonamide (CH_3_SO_2_NH-C_6_H_4_-I), which was prepared from 4-iodoaniline, to afford DSS and TSS in 69 and 15% yields, respectively. It should be noted that the polymerization of 3b was responsible for a diminished yield of TSS as a dark brown spot was observed on the TLC baseline, while the formation of mono-, di-, and tri-substituted products were not observed.

### 3.2 Photophysical properties

**Fig. 2:**
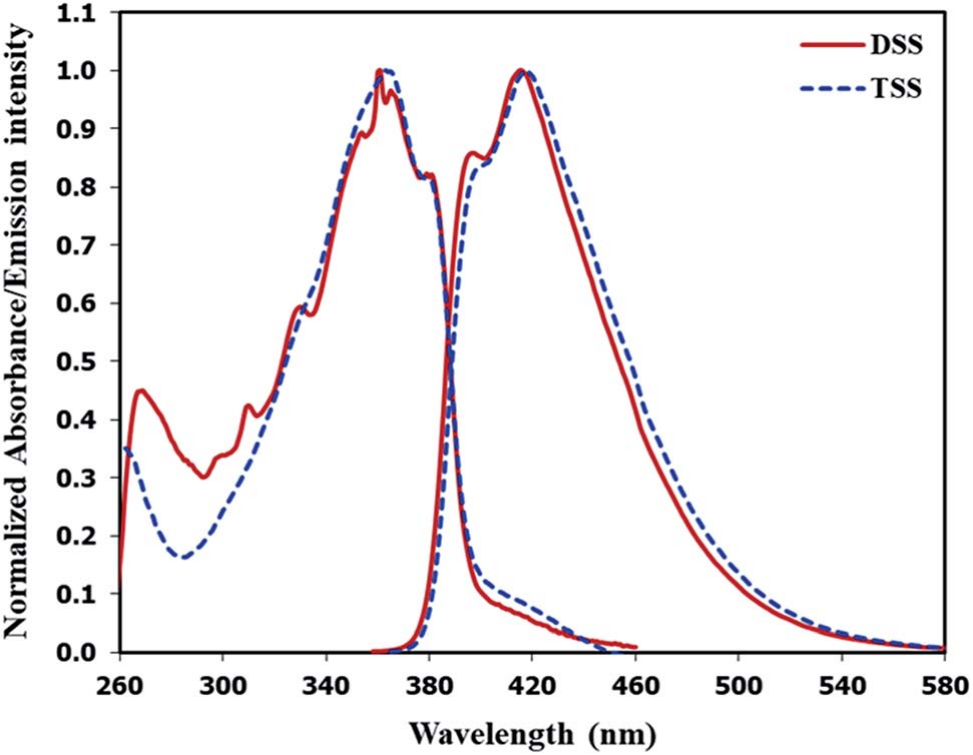
Normalized absorption and emission spectra of DSS and TSS in DMSO.

The UV-Vis absorption and emission spectra of DSS and TSS in DMSO are shown in Fig. 2, and the photophysical properties are summarized in Table 1. The maximum wavelengths of absorption and emission for both compounds were quite similar due to a relatively identical π-conjugated system between phenylacetylene and spirobifluorene units. However, the molar extinction coefficient for TSS is significantly higher than that of DSS because of a higher number of phenylacetylene units. The fluorescence quantum yield of DSS is significantly higher than that of TSS while their fluorescent lifetimes are relatively similar. These data suggest that the excited state of TSS has a higher tendency to lose energy *via* non-radiative proton transfer with DMSO.

**Table 1:** Photophysical properties of DSS and TSS in DMSO

Cmpd.	*λ*_abs_ (nm)	*ε* (M^−1^cm^−1^)	*λ*_em_ a (nm)	*Φ*_f_ b	*τ* c (ns)
DSS	361	26 900	415	0.85	0.74
TSS	363	45 400	417	0.55	0.80

a Excited at *λ*_abs_.

b Absolute fluorescence quantum yield.

c Fluorescence lifetime.

### 3.3 Solvent effect

The fluorescence spectra of DSS and TSS in various solvents are shown in Fig. S1.† The data suggested that DSS and TSS have good solubility in most solvents. In non-polar solvent like hexane and highly polar solvent like DMSO and water, a bathochromic shift and lower emission signals were detected. This could be rationale by their poor solubility in hexane and water or stabilization of excited states by DMSO. Even though the fluorescent signals are lower in DMSO compared to ethanol, methanol or acetonitrile, its high boiling point and low evaporating rate make it a suitable solvent for stock solution. Since the Hg(ii) sensing applications are usually conducted in aqueous media, the optimal ratio of water and DMSO was investigated, and the results are shown in Fig. S2.† The fluorescence intensities of both compounds decreased upon the increment of water content, probably due to some aggregation or precipitation. Based on their emission intensity and UV-Vis absorbance in Fig. S3,† 50% and 30% seem like thresholds of water contents for further sensing studies of DSS and TSS, respectively.

### 3.4 Metal ion sensing studies

**Fig. 3:**
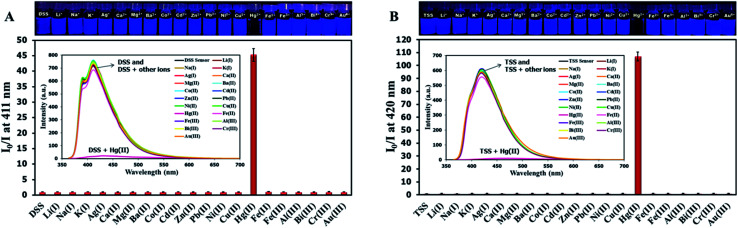
(A) Fluorescence responses of DSS (10 μM) and (B) TSS (10 μM) towards various metal ions (100 μM).

**Fig. 4:**
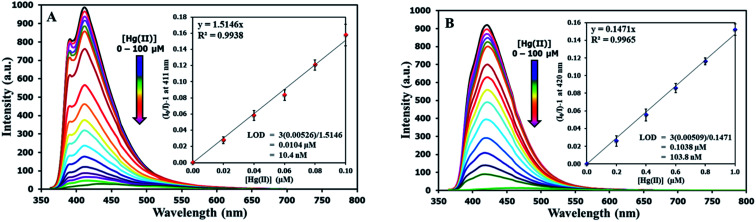
(A) Fluorescence responses of DSS (10 μM) and (B) TSS (10 μM) towards various concentrations of Hg(ii).

The selectivity screening was performed using stock solutions of DSS and TSS in DMSO diluted with HEPES buffer pH 7.0 and DMSO to achieve 10 μM solution of sensors. These solutions were treated with 10 mole equivalents of 20 metal ions including Li(i), Na(i), K(i), Ag(i), Ca(ii), Mg(ii), Ba(ii), Co(ii), Cd(ii), Zn(ii), Pb(ii), Ni(ii), Cu(ii), Hg(ii), Fe(ii), Fe(iii), Al(iii), Bi(iii), Cr(iii), and Au(iii). The results in Fig. 3 show that DSS and TSS have excellent selectivity towards Hg(ii) ion with a 45- and 107-folds fluorescence quenching, respectively. The quantitative determination of Hg(ii) was studied by fluorescence titration experiments. Linear plots were obtained in the Hg(ii) concentrations between 0 to 0.1 μM for DSS and 0 to 1 μM for TSS (Fig. 4). The detection limits for Hg(ii) at three-time noise were 10.4 nM for DSS and 103.8 nM for TSS. In addition, the interfering effect of foreign ions at 1 mM (10 times concentration of Hg(ii)) was studied, and the results indicated that only Fe(ii) can interfere the detection of Hg(ii) (Fig. S4†). A competitive absorption mechanism was proposed since the UV-Vis spectra of Fe(ii) completely overlapped with those of DSS and TSS (Fig. S5†).

### 3.5 Effect of pH

Because DSS and TSS contain acidic –NH groups, it is important to study the effect of pH on their fluorescence signals and sensitivity towards Hg(ii). Data in Fig. S6† indicated that the original fluorescence signals of DSS and TSS were steady under acidic to neutral pH ranges and gradually decreased in basic media. Upon addition of Hg(ii), the signals were decreased at all pHs, however, the quenching effect was most pronounced between 6.5 to 7.5. Therefore, the optimal working pH was selected at 7.0. The pH effect also provides a hint on the sensing mechanism which will be explained later.

### 3.6 Mechanism of sensing

**Fig. 5:**
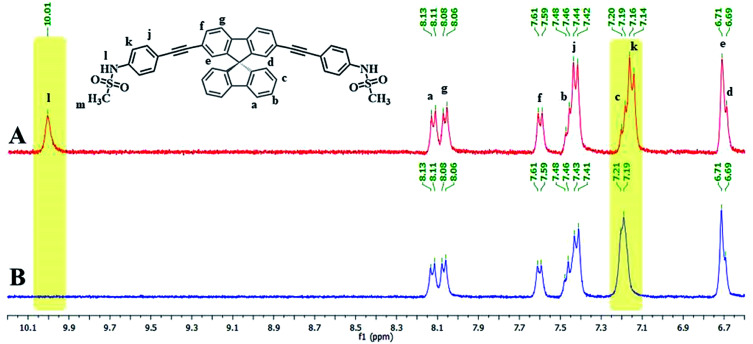
(A) ^1^H-NMR spectra of DSS and (B) DSS + Hg(OAc)_2_ (1.0 eq.) in D6-DMSO.

Since the emission signals of both DSS and TSS were lowered under basic pHs, it is likely that the sensing mechanism may involve the deprotonation. For the investigation of sensing mechanism, we first examined the ^1^H-NMR spectra of DSS in D6-DMSO before and after the addition of 1 equivalent of Hg(ii), and found that the –NH signal disappeared and the signal for the nearby aromatic –CH shifted slightly downfield when Hg(ii) was added (Fig. 5). We postulated that the coordination between the sulfonamide and Hg(ii) could promote a deprotonation of the –NH group at neutral pH. The addition of EDTA to DSS–Hg(ii) and TSS–Hg(ii) mixtures could restore the emission signals, which were completely recovered when the Hg(ii)-EDTA stoichiometry reached 1 : 1 (Fig. S7†). In addition, lower absorbance and shifted baseline were observed in the UV-Vis spectra of DSS–Hg(ii), along with a Tyndall effect (Fig. S8†). These results confirmed that the sensing mechanism involved a coordination between the sulfonamide group and Hg(ii) that lead to an aggregation of such complex presumably *via* the face-to-face stacking of the spirobifluorene cores.^30^ This static binding quenching mechanism was ultimately proven by Stern–Volmer plots at different temperatures (Fig. S9†). The decreases in quenching efficiencies (lower *K*_SV_) at the higher temperature suggested that the formation of Hg(ii) complex is responsible for signals quenching.

### 3.7 Quantitative analysis of Hg(ii) in water samples

To demonstrate the use of DSS and TSS as sensors for Hg(ii) in water, we selected 3 sources of water samples, spiked them with Hg(ii), and analyzed the amount of Hg(ii) using traditional ICP-OES technique and our sensors. The analysis results in Table 2 showed a good recovery of Hg(ii) in each sample, while the statistical results in Table S1† indicated an insignificant difference between data from ICP-OES and our method (*p*-value > 0.05).

**Table 2:** Analytical results of Hg(ii) in water samples (*n* = 3)

Samples	Hg(ii) added (ppm)	ICP-OES		DSS		TSS	
		Hg(ii) found (ppm)	Recovery (%)	Hg(ii) found (ppm)	Recovery (%)	Hg(ii) found (ppm)	Recovery (%)
Rain water	0.5	0.504 ± 0.032	101	0.482 ± 0.052	96	0.474 ± 0.040	95
	1.0	1.118 ± 0.022	112	1.041 ± 0.048	104	1.101 ± 0.085	110
Tap water	0.5	0.513 ± 0.030	103	0.506 ± 0.045	101	0.512 ± 0.067	102
	1.0	1.185 ± 0.036	118	1.055 ± 0.058	105	1.116 ± 0.099	112
Bottled water	0.5	0.571 ± 0.011	114	0.547 ± 0.037	109	0.565 ± 0.032	113
	1.0	1.082 ± 0.019	108	1.022 ± 0.028	102	1.024 ± 0.071	102

### 3.8 Detection of l-glutathione

**Fig. 6:**
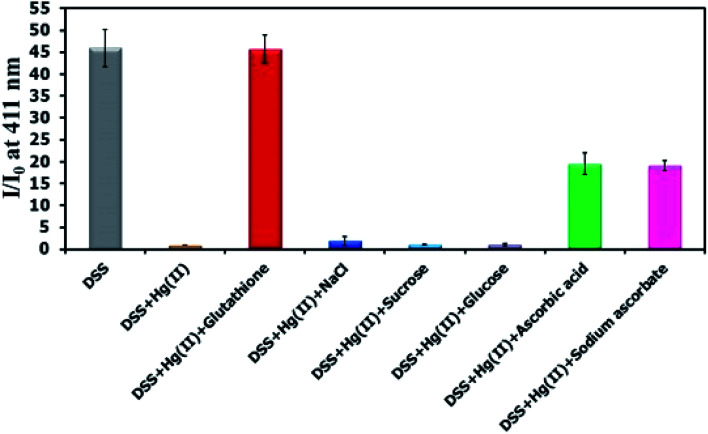
Fluorescence response of DSS (10 μM) with Hg(ii) (100 μM) and other beverage ingredients (300 μM).

**Fig. 7:**
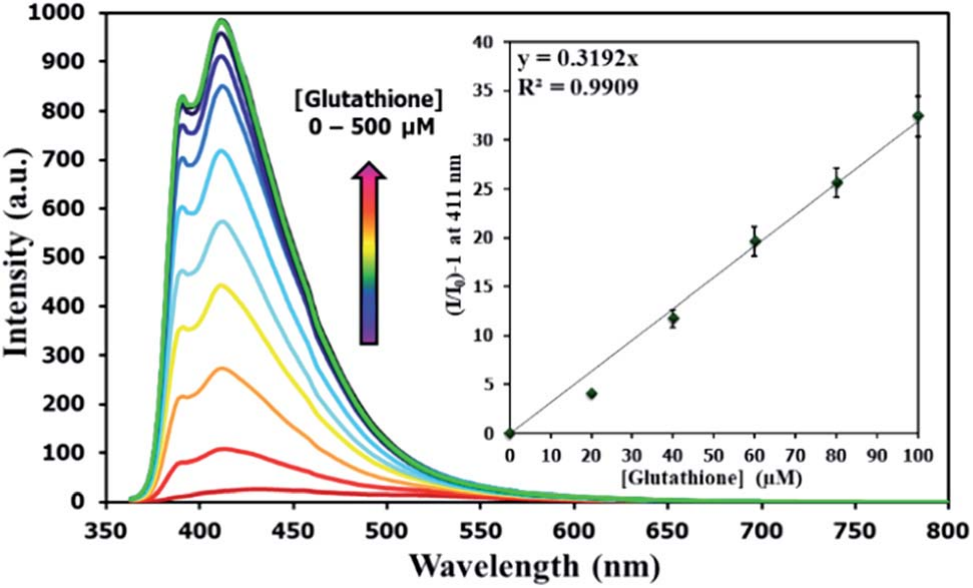
Fluorescence responses of DSS (10 μM) with Hg(ii) (100 μM) upon addition of l-glutathione.

There have been several reports on specific binding between Hg(ii) and l-glutathione, which is a biologically important antioxidant and a major component in food supplements and cosmetics. In our study, we tested the ability of l-glutathione and other food ingredients to restore the fluorescence signal of DSS, and found that the DSS–Hg(ii) complex can exhibit a selective fluorescence turn-on by l-glutathione (Fig. 6, S10†). Ascorbic acid and its sodium salt, which are known to coordinate with Hg(ii), can also restore the fluorescence signal, albeit in lower levels. However, the amount of ascorbic acid in most samples is usually 10-folds lower than l-glutathione. Quantitative analysis revealed a detection limit of about 0.6 μM or approximately 200 ppb of l-glutathione (Fig. 7). The applications of DSS for analyses of l-glutathione on three supplement beverages showed good agreements between the labeled and experimental contents (Table 3).

**Table 3:** Analytical results of l-glutathione in three commercial beverages (*n* = 3)

Samples	Labeled content (%)	Analyzed by DSS–Hg(ii)	
		Found (%)	Recovery (%)
1	0.05	0.0512 ± 0.0042	102
2	0.10	0.1102 ± 0.0090	110
3	0.30	0.2946 ± 0.0128	98

## 4. Conclusion

In summary, we successfully synthesized two derivatives of spirobifluorene containing methanesulfonamide groups. Both compounds showed a selective fluorescence quenching by Hg(ii) *via* complexation and aggregation-caused quenching. The detection limits for Hg(ii) were 10.4 nM and 103.8 nM for the derivatives bearing two and four sulfonamide groups, respectively. One of these mercury complexes was used as a selective turn-on sensor for l-glutathione with a detection limit of 0.6 μM. A satisfactory quantitative analysis of l-glutathione in three supplement drinks was also demonstrated.

## Conflicts of interest

There are no conflicts to declare.

## Supplementary Material

RA-009-C9RA00004F-s001
