# Supplementary material for: Novel sulfonamidospirobifluorenes as fluorescent sensors for mercury(ii) ion and glutathione
Source: RSC Adv. 2019 Apr 11;9(20):11451–8. doi: 10.1039/c9ra00004f (PMC9063287; doi:10.1039/c9ra00004f)
Supplement: RA-009-C9RA00004F-s001 [file RA-009-C9RA00004F-s001.pdf]

Electronic Supplementary Material (ESI) for RSC Advances.
This journal is © The Royal Society of Chemistry 2019

Supporting Information

Novel Sulfonamido Spirobifluorene as Fluorescent Sensorsfor Mercury(II) Ion and Glutathione

Komthep Silpcharu[a], Mongkol Sukwattanasinitt[a], Paitoon Rashatasakhon[a,*]

[a]Department of Chemistry, Faculty of Science, Chulalongkorn University, Bangkok 10330, Thailand

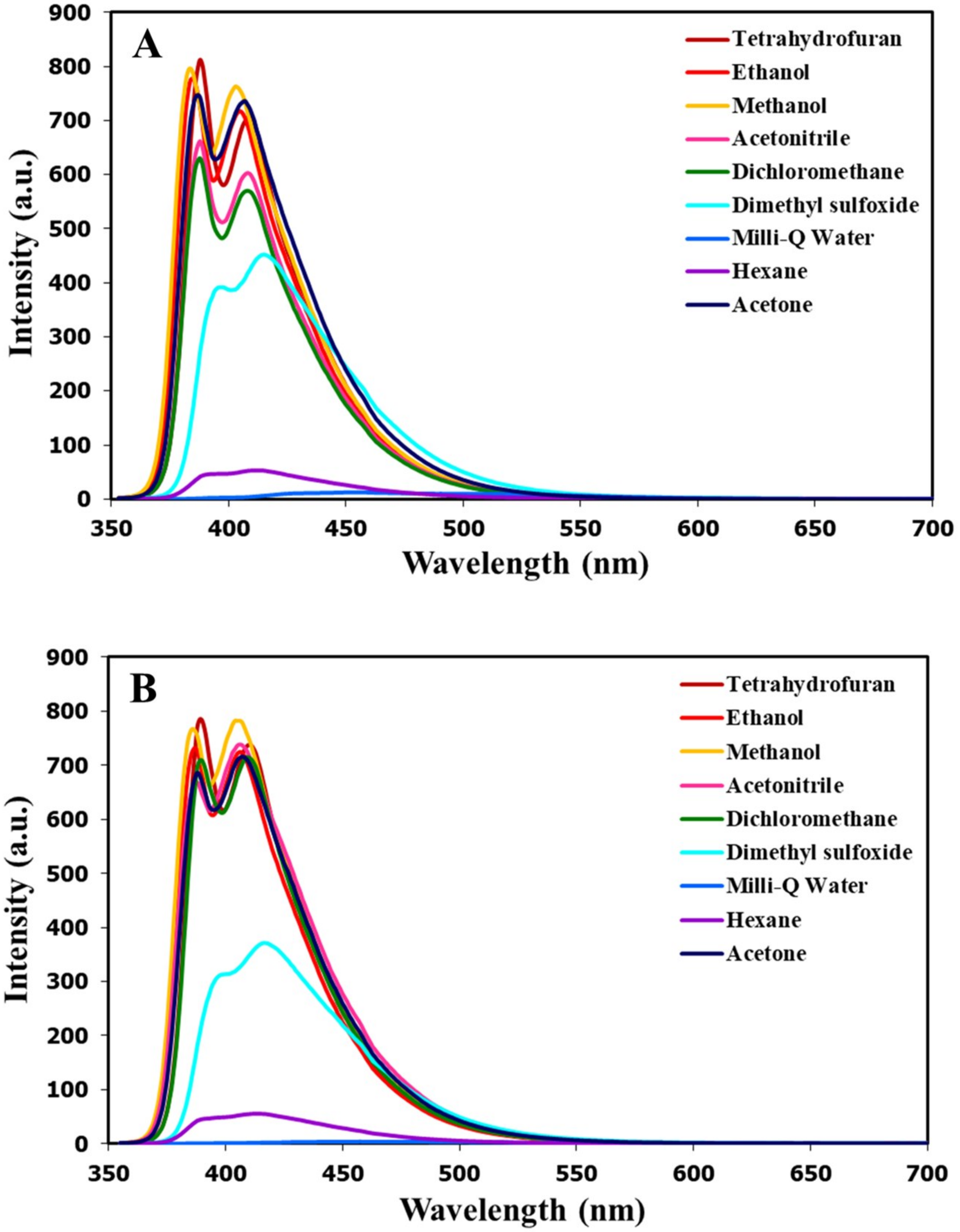

Figure S1. Fluorescence emission spectra of (A) **DSS** (10µM)and (B)**TSS**(10µM) in various solvents

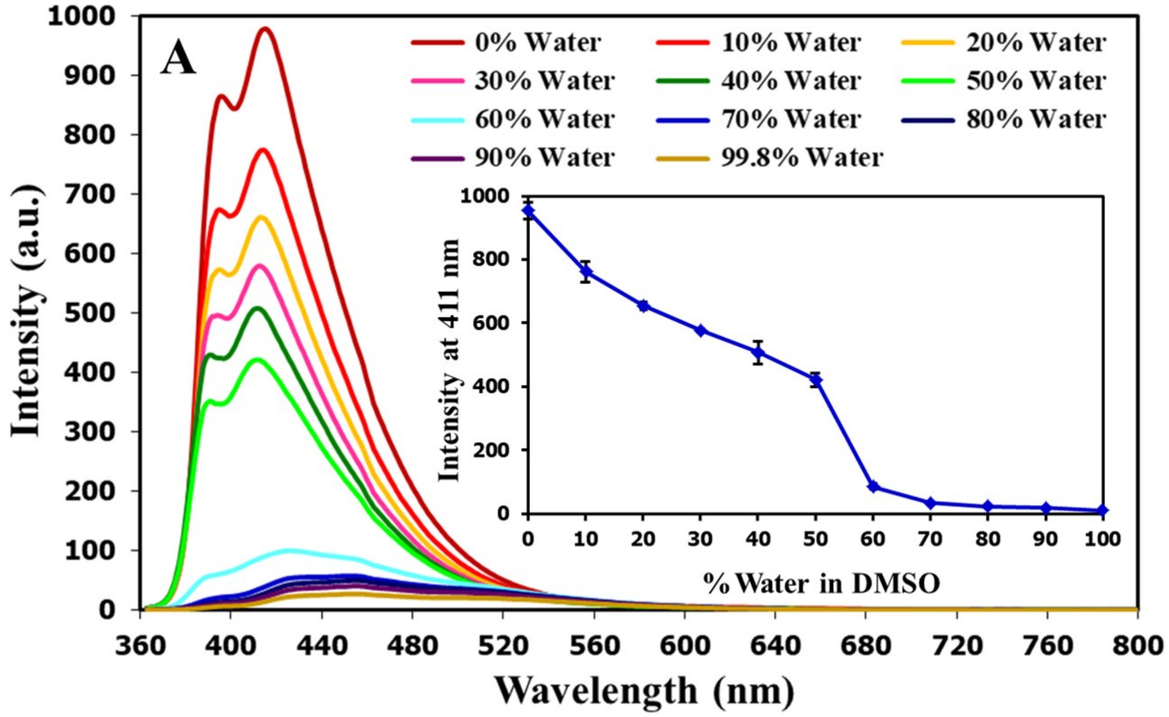

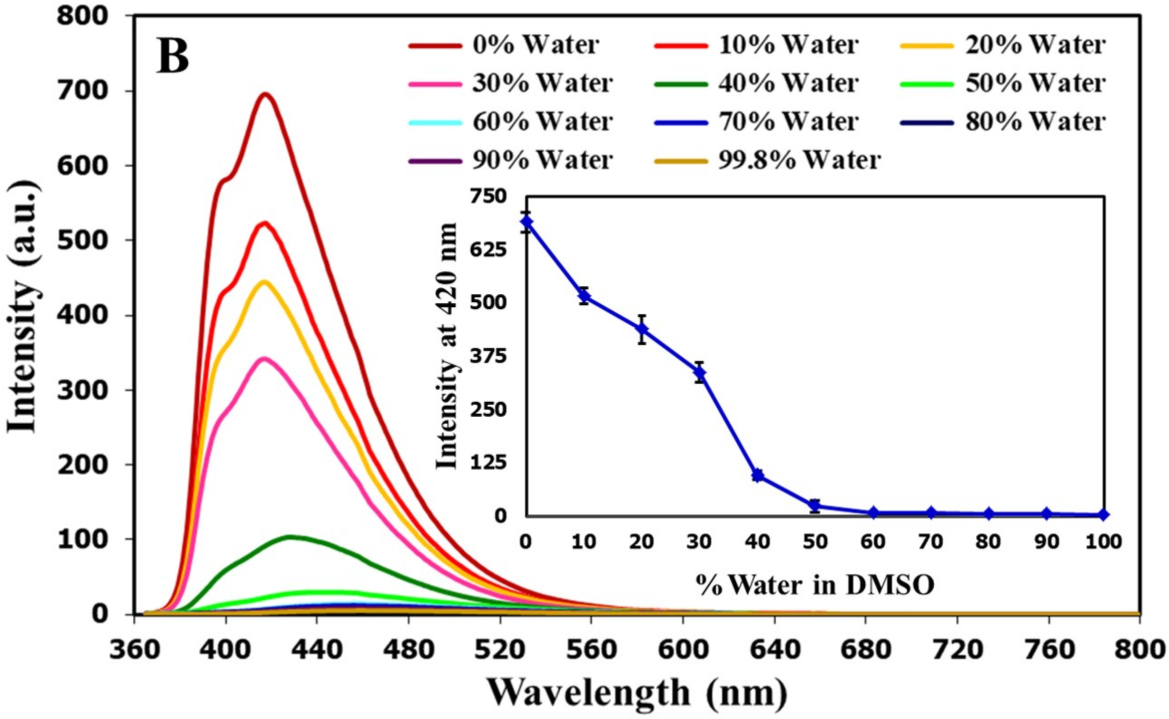

Figure S2. Fluorescence spectra of (A) **DSS**(10μM) and (B)**TSS**(10μM) in various water/DMSO ratios.

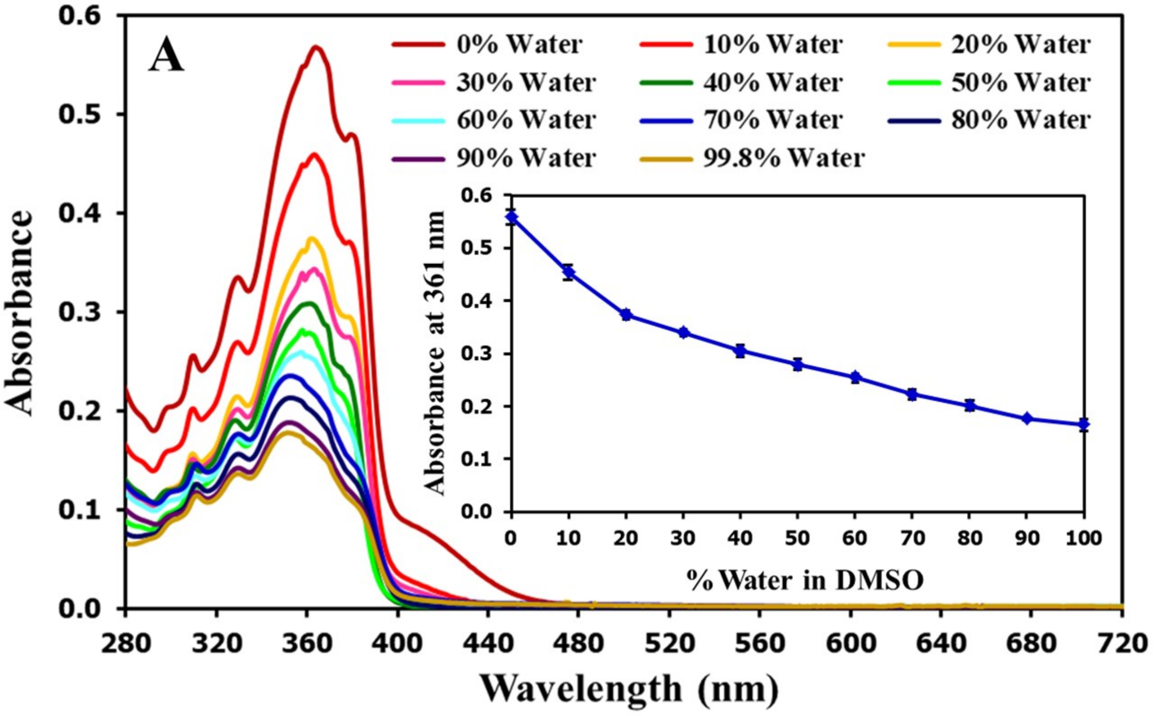

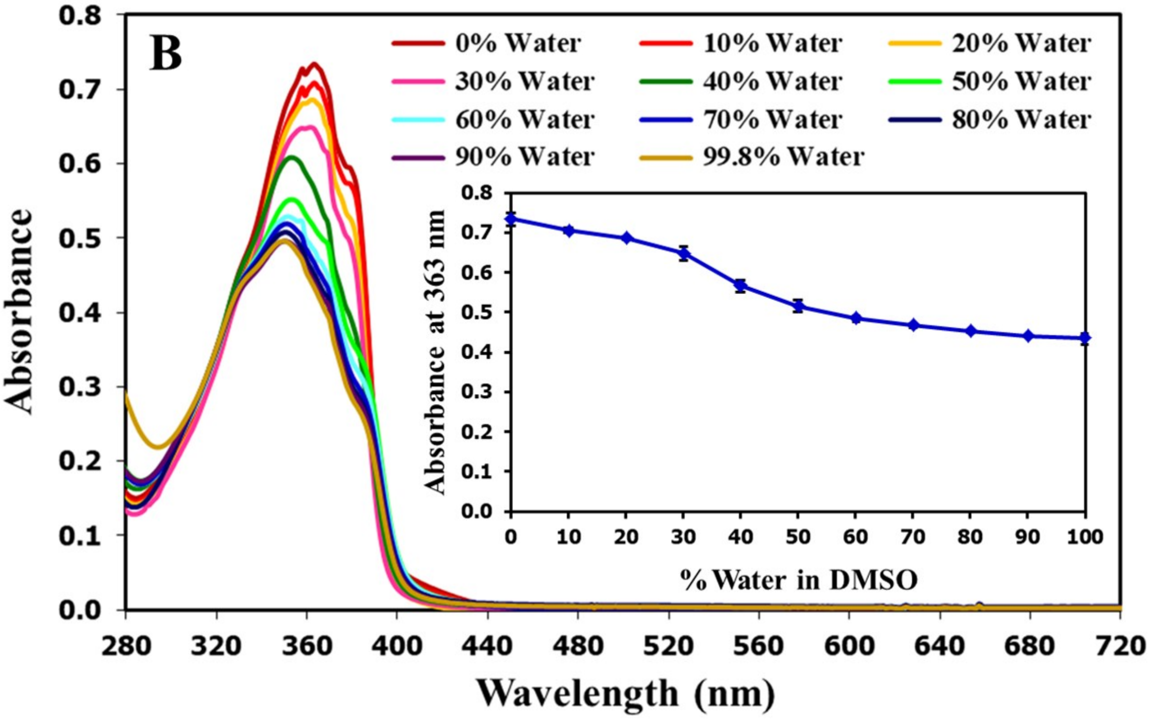

Figure S3. Absorption spectra of (A) **DSS**(10μM) and (B) **TSS**(10μM) in various water/DMSO ratios.

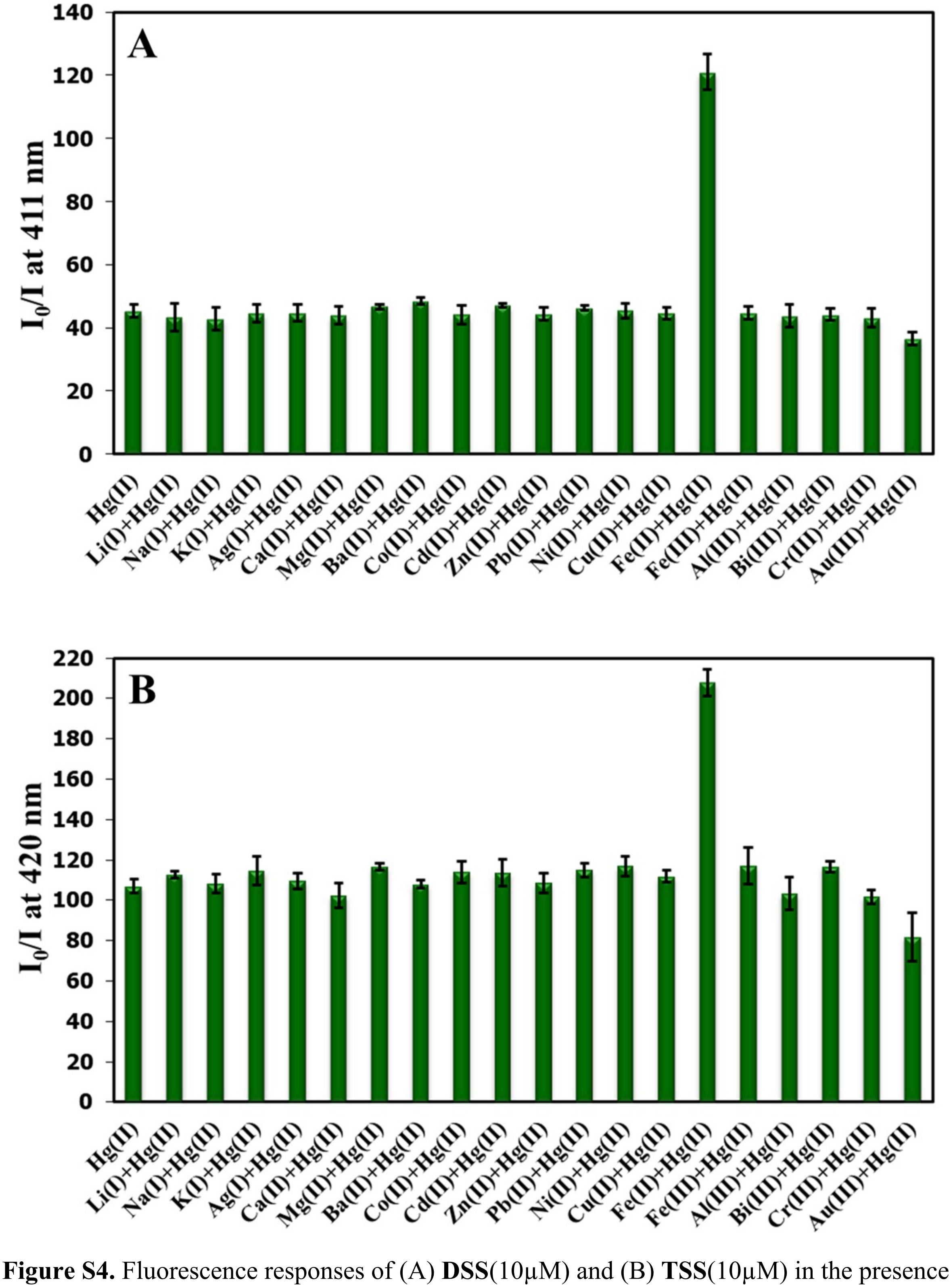

Figure S4. Fluorescence responses of (A) **DSS**(10μM) and (B) **TSS**(10μM) in the presence of Hg(II) (100μM) andforeign ions (1000μM)

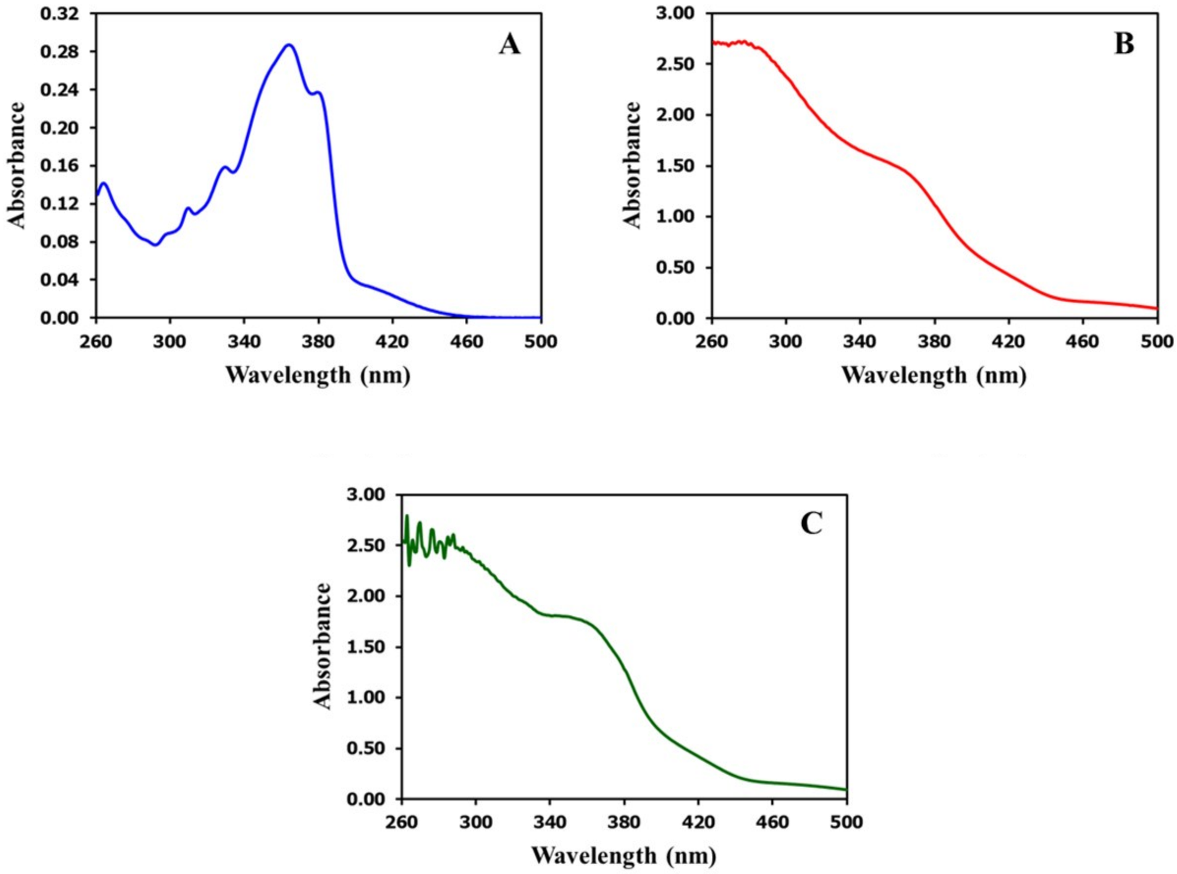

Figure S5. UV spectra of (A) 10 μM **DSS**, (B) 1000 μM $Fe(OAc)_2$, and (C) 10 μM **DSS** + 1000 μM $Fe(OAc)_2$in 50% of 10 mM HEPES buffer in DMSO, pH 7.0.

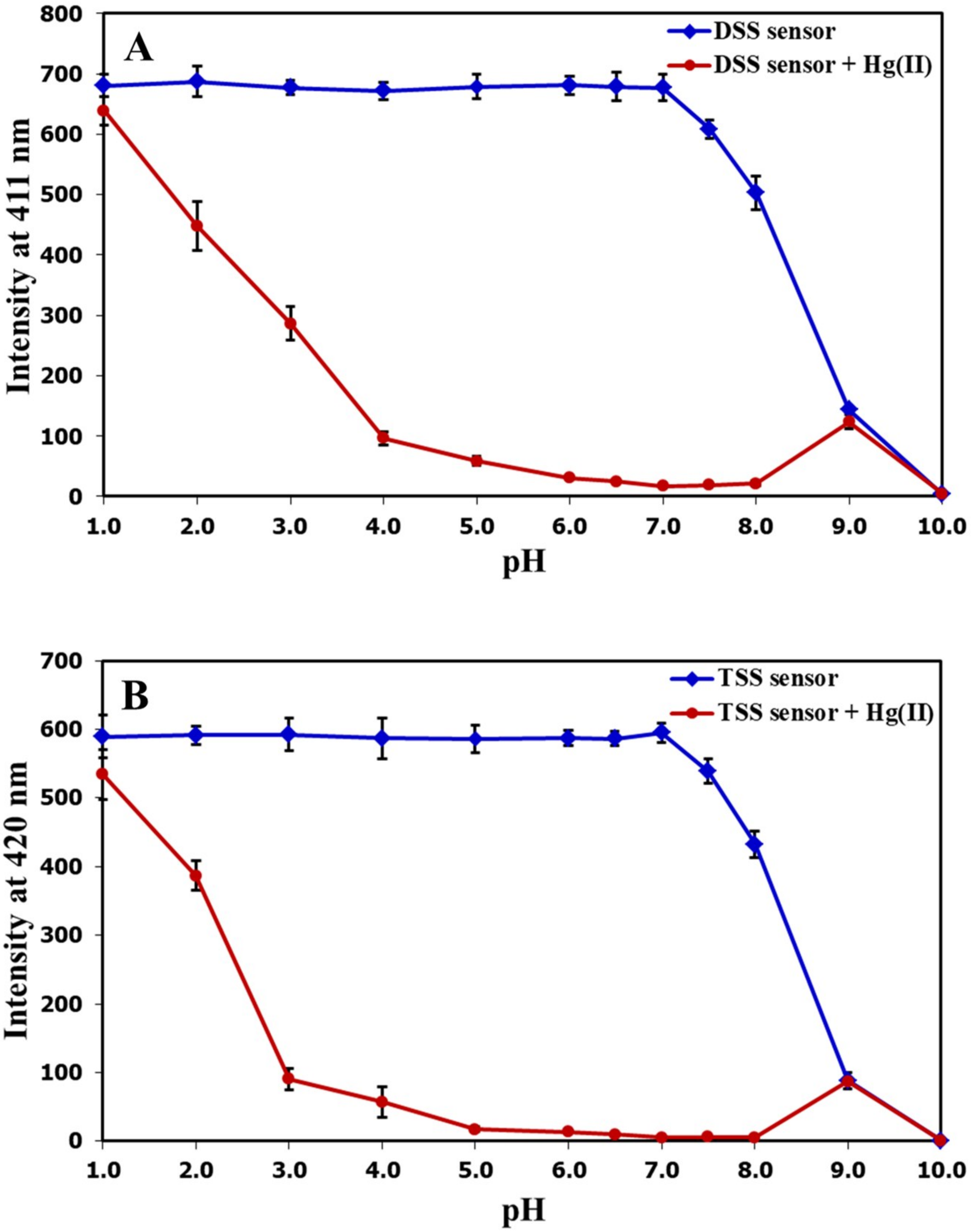

Figure S6. pH effects on emission intensities of (A) **DSS**(10μM) and (B)**TSS** (10μM) before and after addition of Hg(II) (100μM)

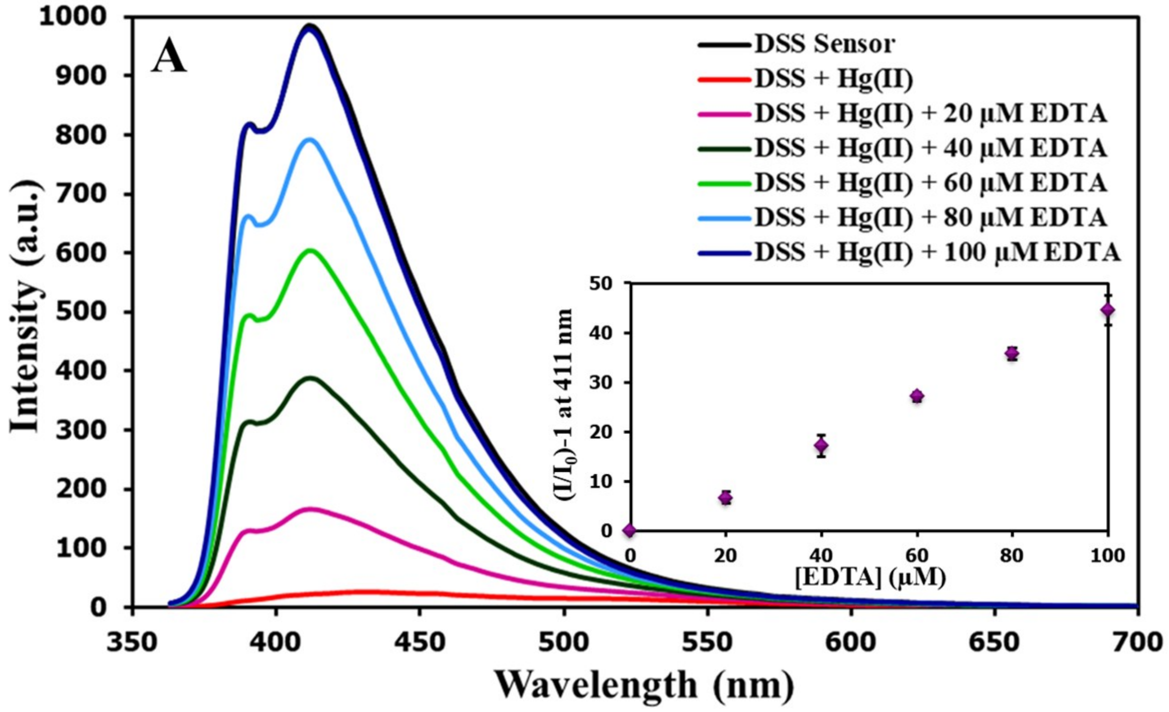

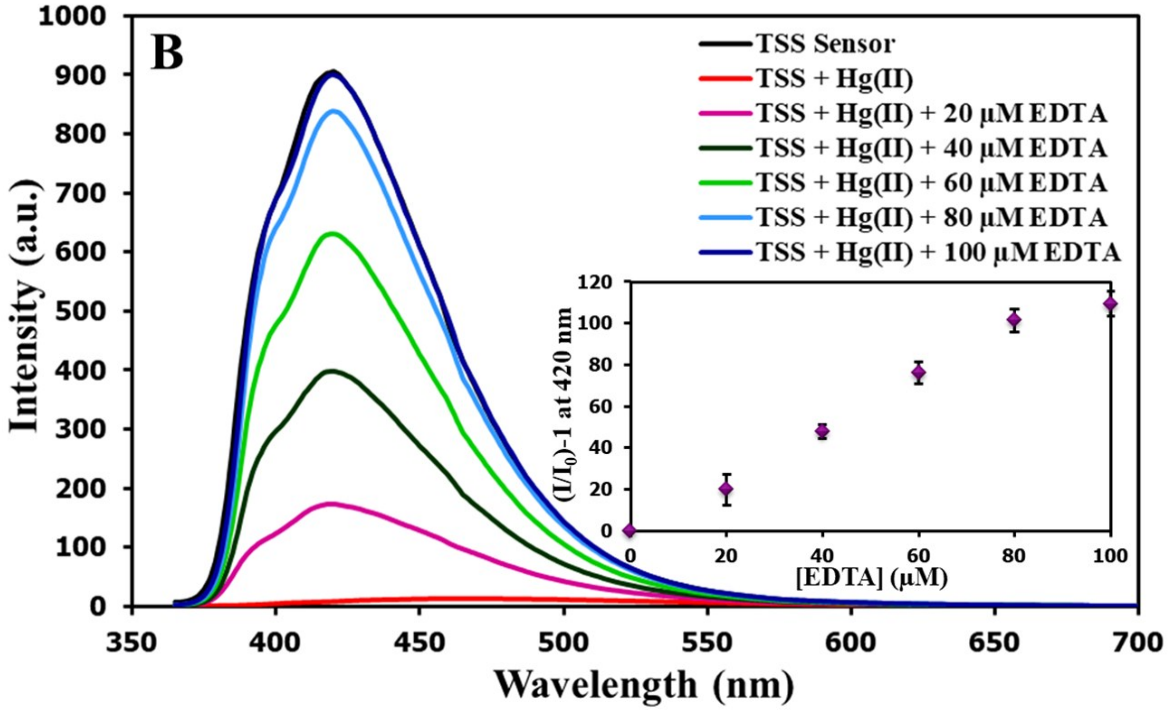

Figure S7. Fluorescence spectra of (A) **DSS** (10µM) and (B) **TSS** (10µM) with Hg(II) (100µM) and various amounts of EDTA

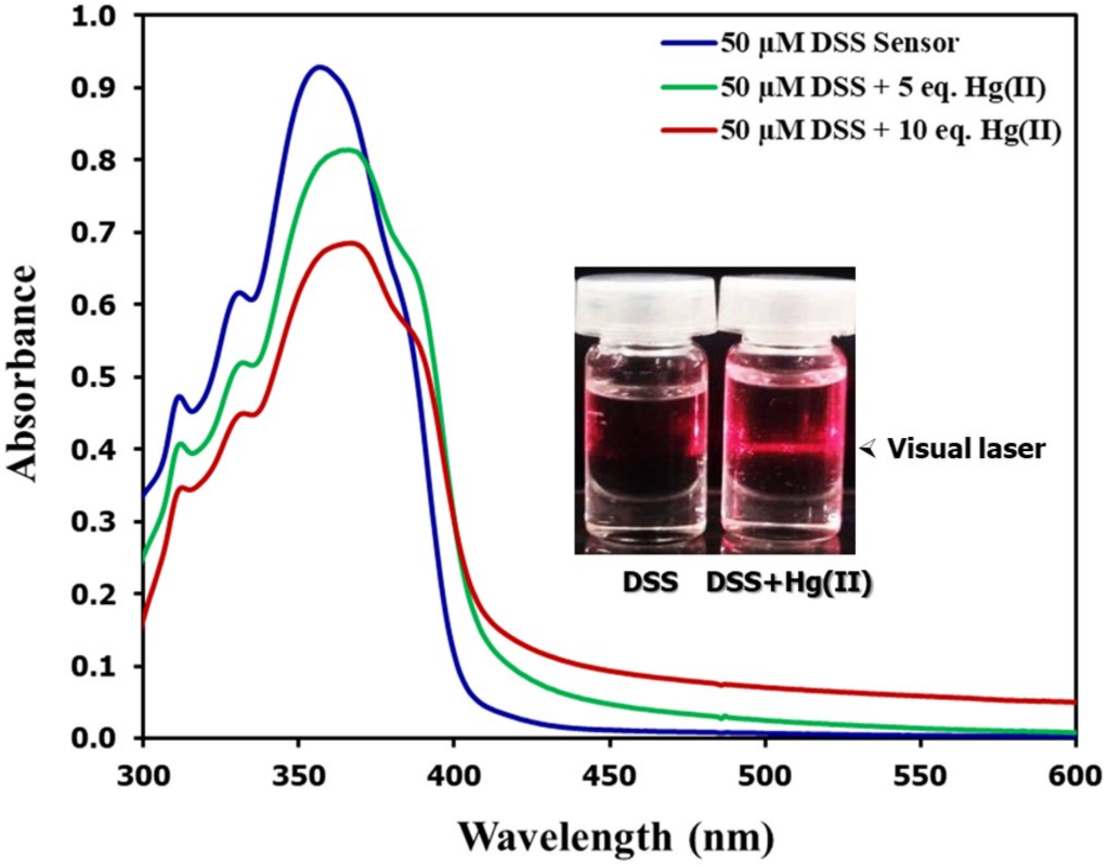

Figure S8. UV spectra for **DSS** before and after addition of $Hg(OAc)_2$ (5 and 10 eq.) in 50% of 10 mM HEPES buffer in DMSO, pH 7.0. Inset is the photographed image showing the Tyndall effect.

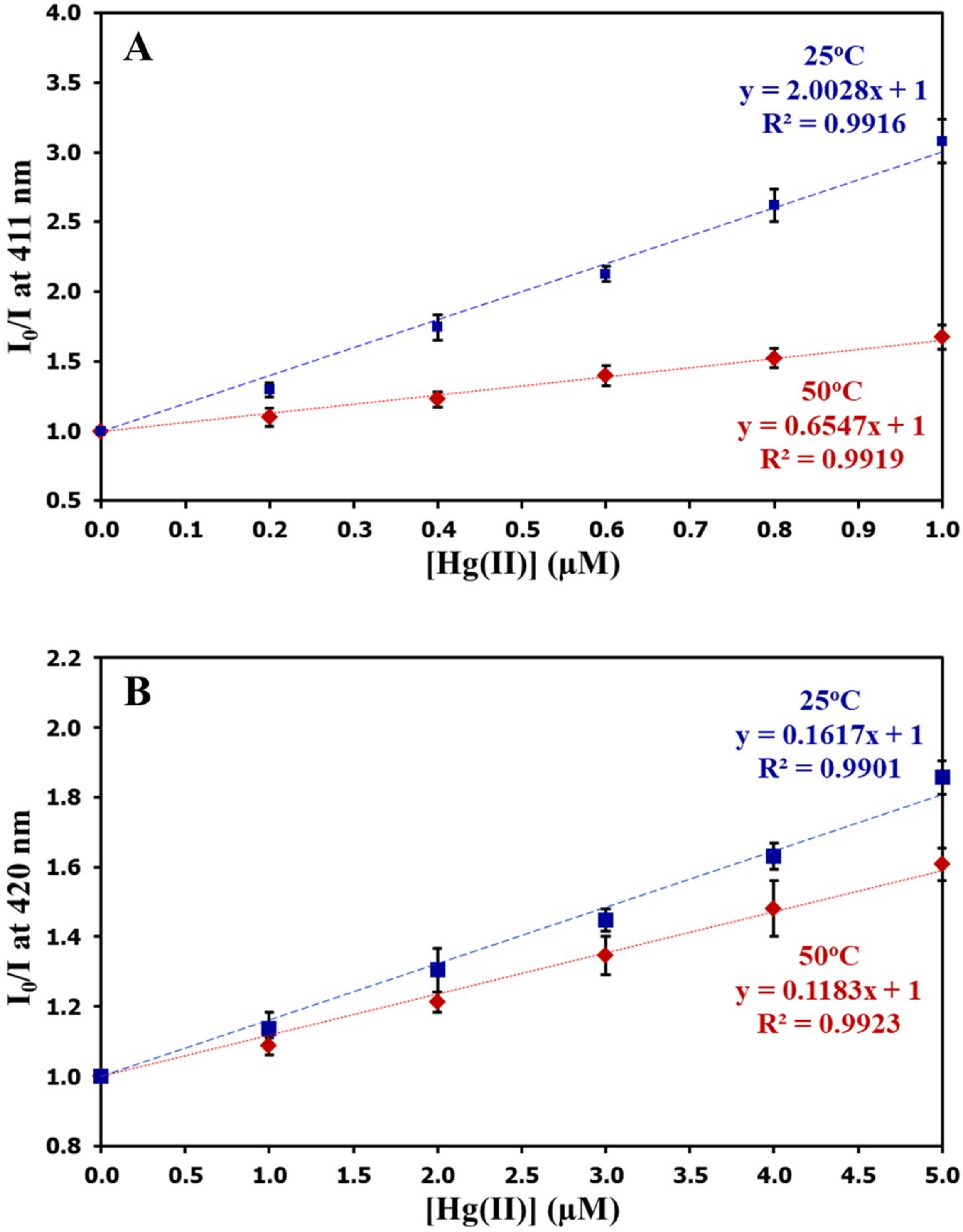

Figure S9. Stern-Volmer plots for (A) **DSS** (10μM) and (B)**TSS** (10μM) at 25 and 50°C.

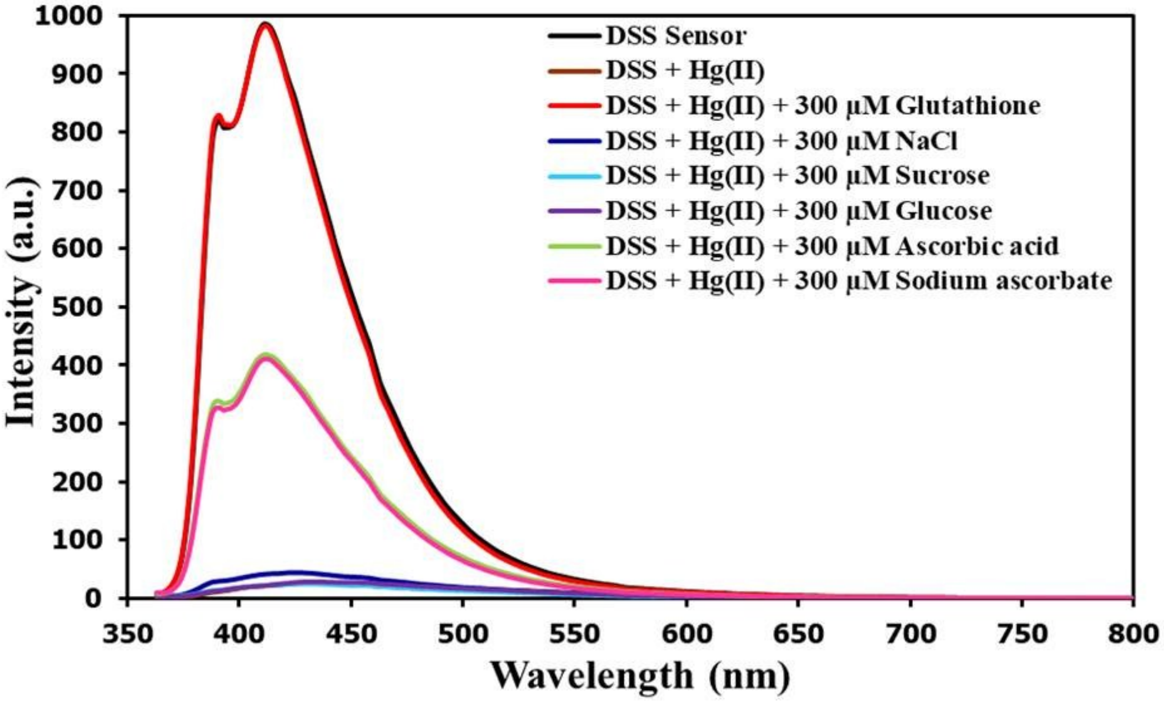

Figure S10. Fluorescence spectra of mixtures between **DSS** (10μM) and Hg(II) (100 μM) in the presence of type of some beverage ingredients (300 μM).

Table S1. Statistic data

Quantitative Analysis of DSS Sensor

Type	[Hg(II)] (ppm)	[Hg(II)] in sample (ppm)				
		1st	2nd	3rd	Average	SD
**Bottled water**	0.00	0.029	-0.020	-0.020	-0.003	0.028
	0.50	0.517	0.588	0.536	0.547	0.037
	1.00	1.000	1.053	1.012	1.022	0.028
**Rain water**	0.00	0.032	-0.020	-0.021	-0.003	0.031
	0.50	0.423	0.523	0.500	0.482	0.052
	1.00	1.096	1.017	1.009	1.041	0.048
**Tap water**	0.00	0.006	-0.005	-0.013	-0.004	0.009
	0.50	0.486	0.557	0.473	0.506	0.045
	1.00	1.122	1.027	1.016	1.055	0.058
P-value		0.12	0.12	0.12	0.13	

Quantitative Analysis of TSS Sensor

Type	[Hg(II)] (ppm)	[Hg(II)] in sample (ppm)				
		1st	2nd	3rd	Average	SD
**Bottled water**	0.00	0.017	-0.011	-0.034	-0.009	0.026
	0.50	0.597	0.532	0.568	0.565	0.032
	1.00	0.971	1.104	0.996	1.024	0.071
**Rain water**	0.00	0.019	0.010	-0.060	-0.011	0.043

	0.50	0.515	0.435	0.471	0.474	0.040
	1.00	1.197	1.034	1.072	1.101	0.085
**Tap water**	0.00	0.009	-0.106	0.071	-0.009	0.090
	0.50	0.576	0.518	0.441	0.512	0.067
	1.00	1.086	1.226	1.036	1.116	0.099
P-value		0.10	0.15	0.15	0.13	

Quantitative Analysis of ICP-OES

**Type**	**[Hg(II)] (ppm)**	**[Hg(II)] in sample (ppm)**			
		**Hg1849**		**Hg1942**	
		**Average**	**SD**	**Average**	**SD**
**Bottled water**	0.00	0.025	0.001	0.027	0.002
	0.50	0.571	0.011	0.559	0.012
	1.00	1.082	0.019	1.071	0.016
**Rain water**	0.00	0.032	0.006	0.040	0.005
	0.50	0.504	0.032	0.500	0.034
	1.00	1.118	0.022	1.096	0.015
**Tap water**	0.00	0.028	0.004	0.033	0.004
	0.50	0.513	0.030	0.507	0.033
	1.00	1.185	0.036	1.173	0.031
P-value		0.09		0.09	

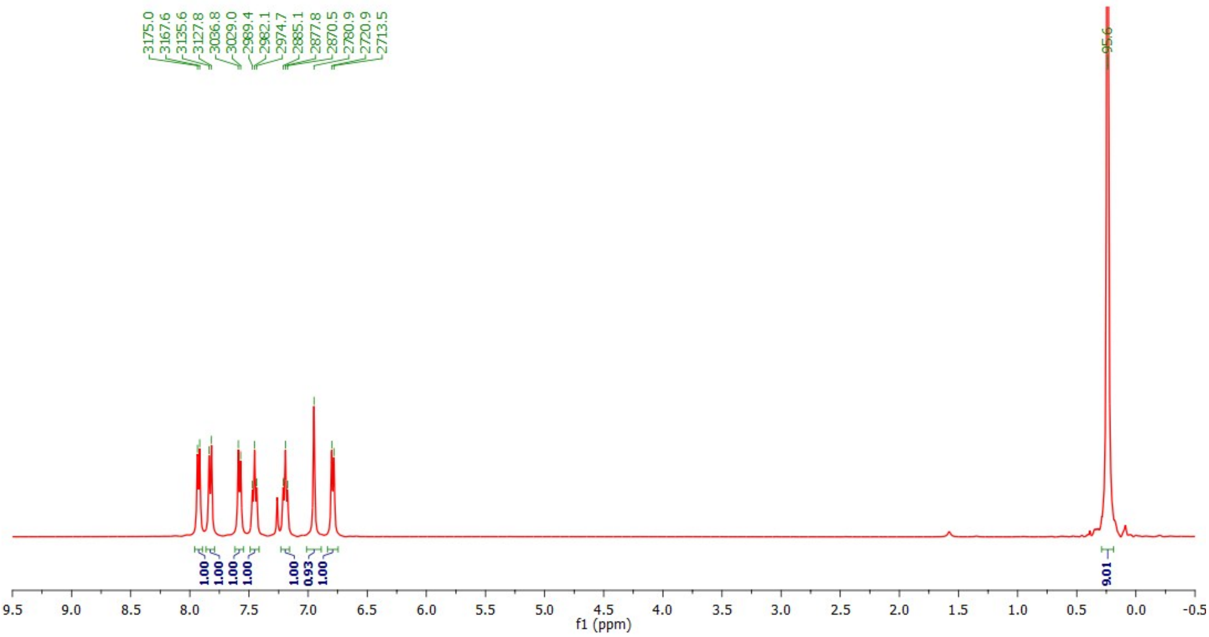

Figure S11.^{1}H-NMR spectrum of compound **2a**.

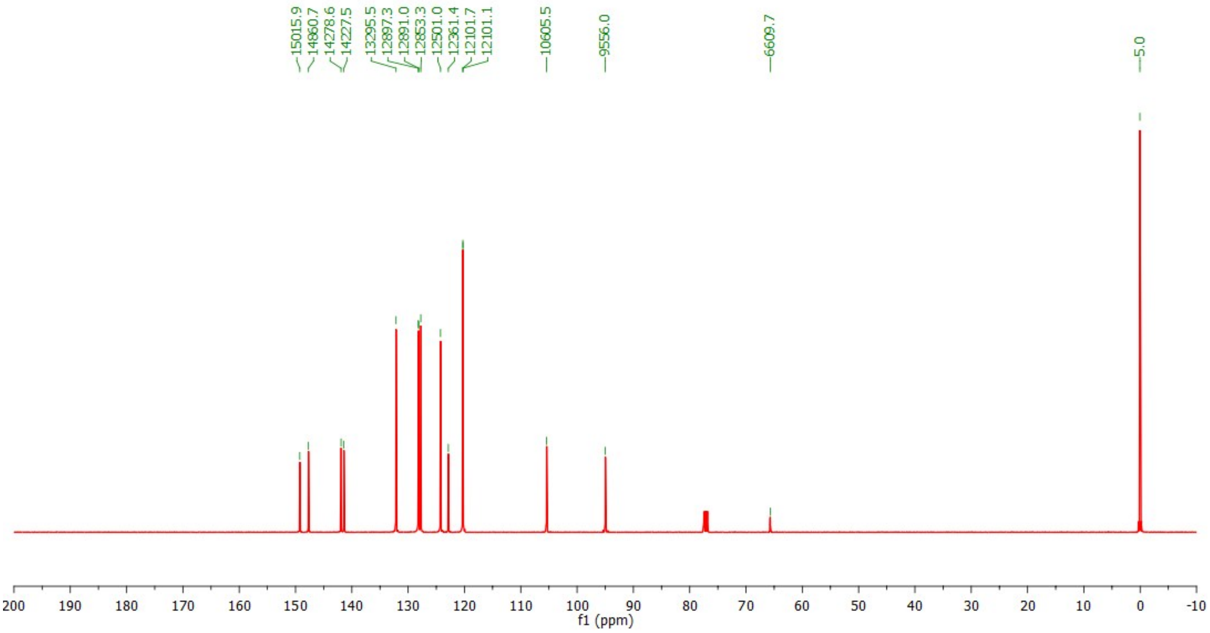

Figure S12.^{13}C-NMR spectrum of compound **2a**.

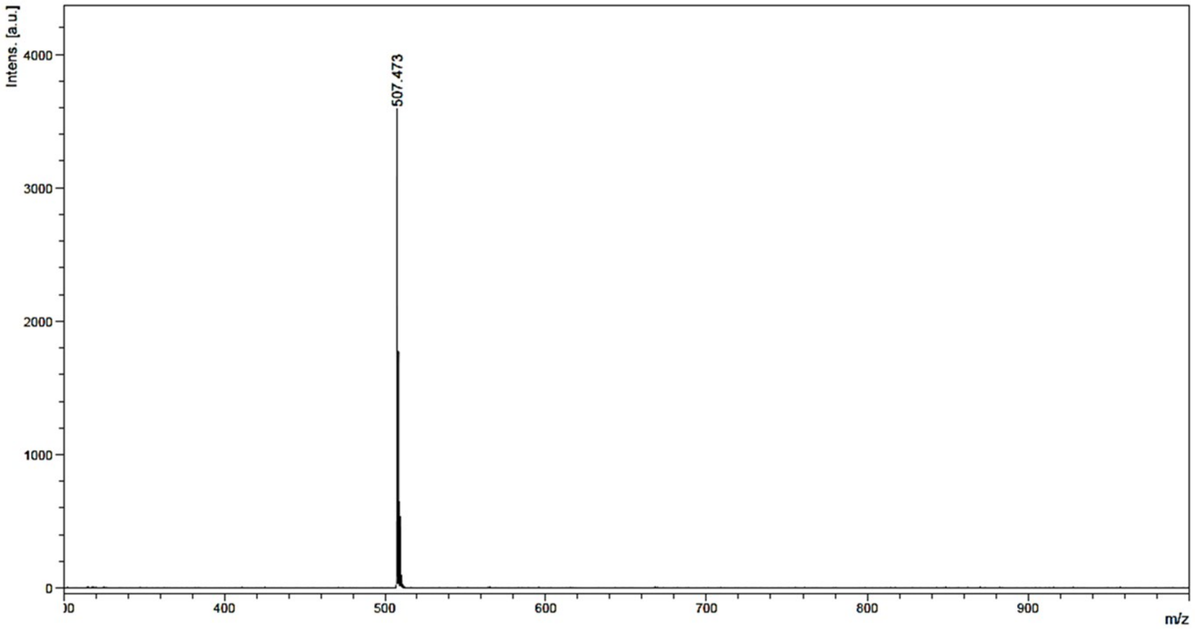

Figure S13. Mass spectrum of compound **2a**.

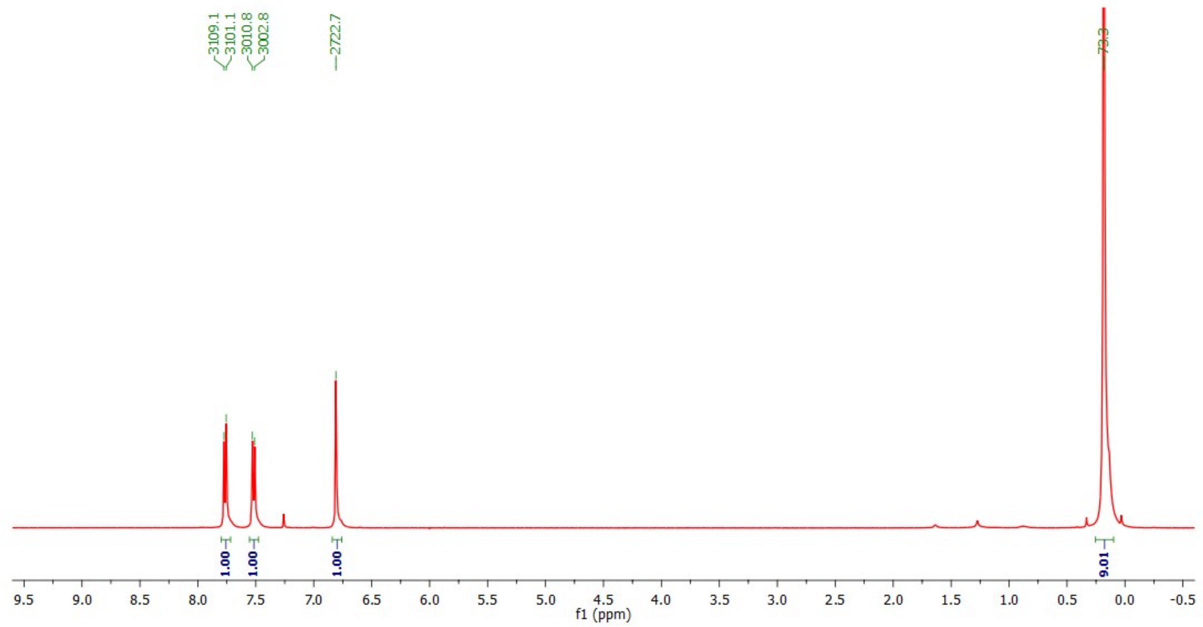

Figure S14.^{1}H-NMR spectrum of compound **2b**.

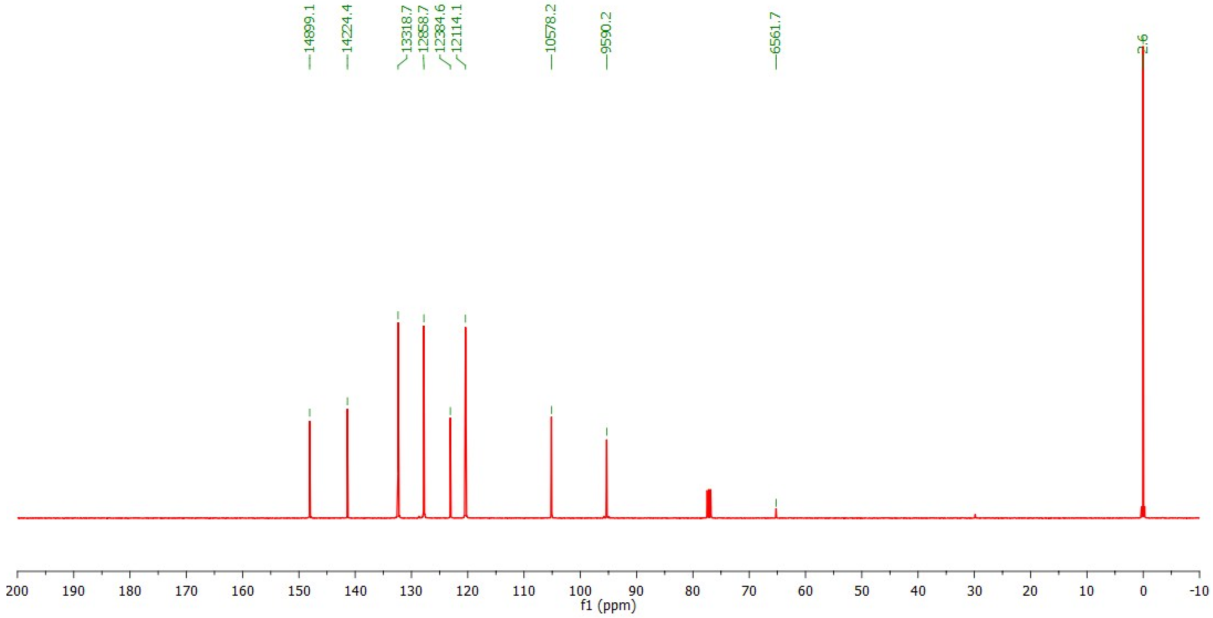

Figure S15.^{13}C-NMR spectrum of compound **2b**.

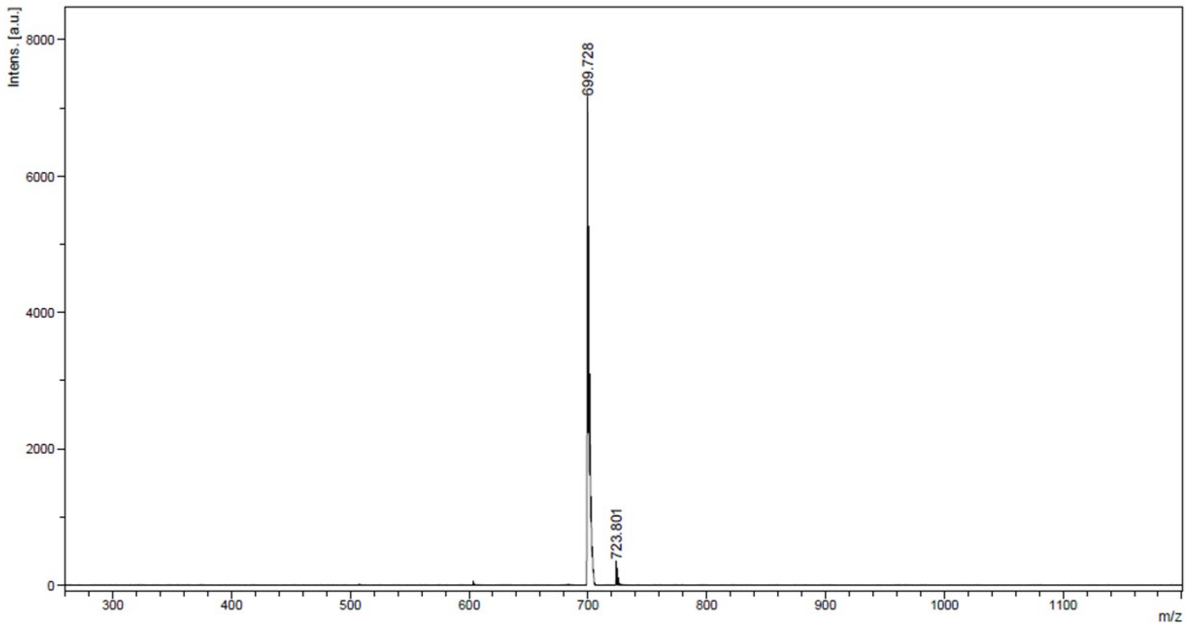

Figure S16. Mass spectrum of compound **2b**.

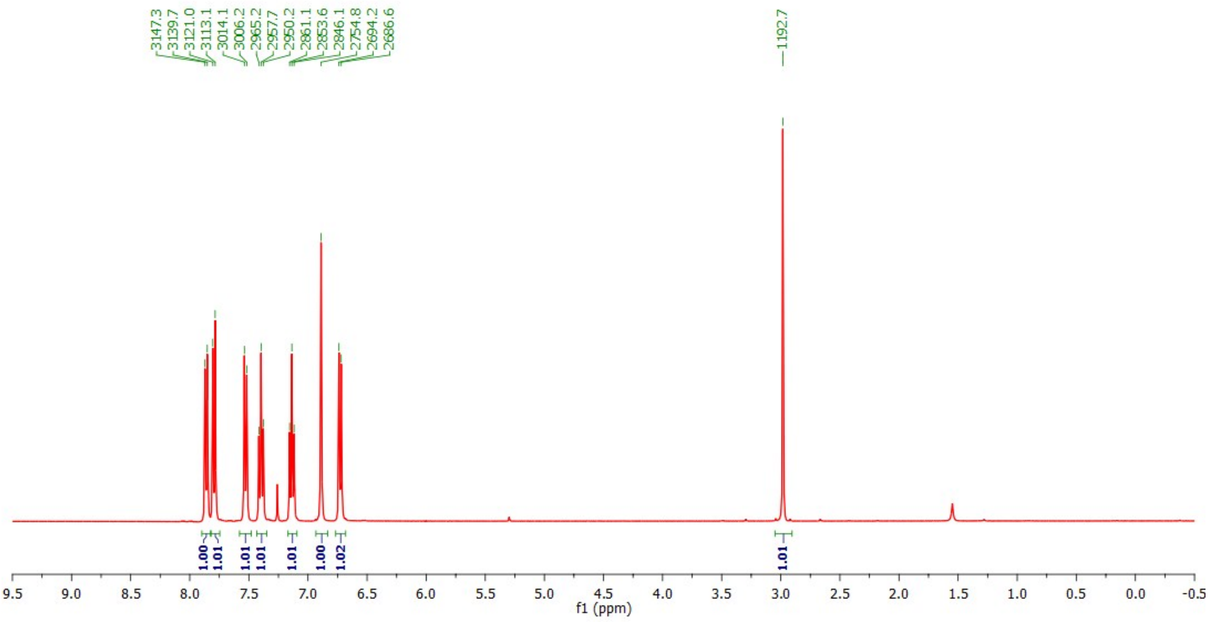

Figure S17. ^{1}H-NMR spectrum of compound **3a**.

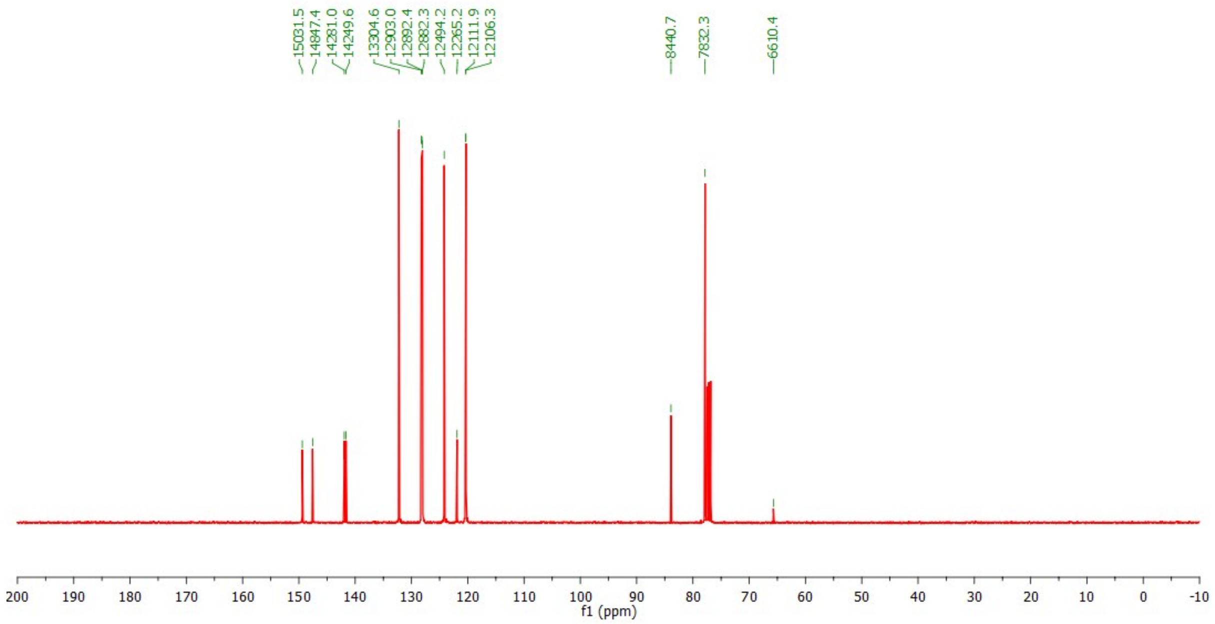

Figure S18. ^{13}C-NMR spectrum of compound **3a**.

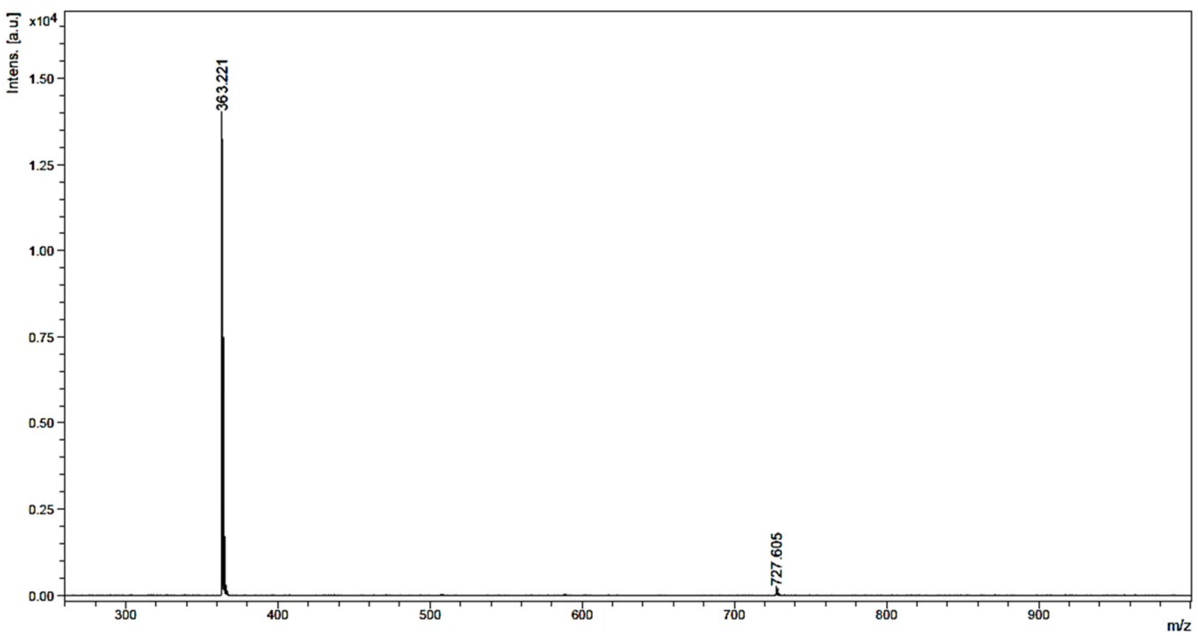

Figure S19. Mass spectrum of compound **3a**.

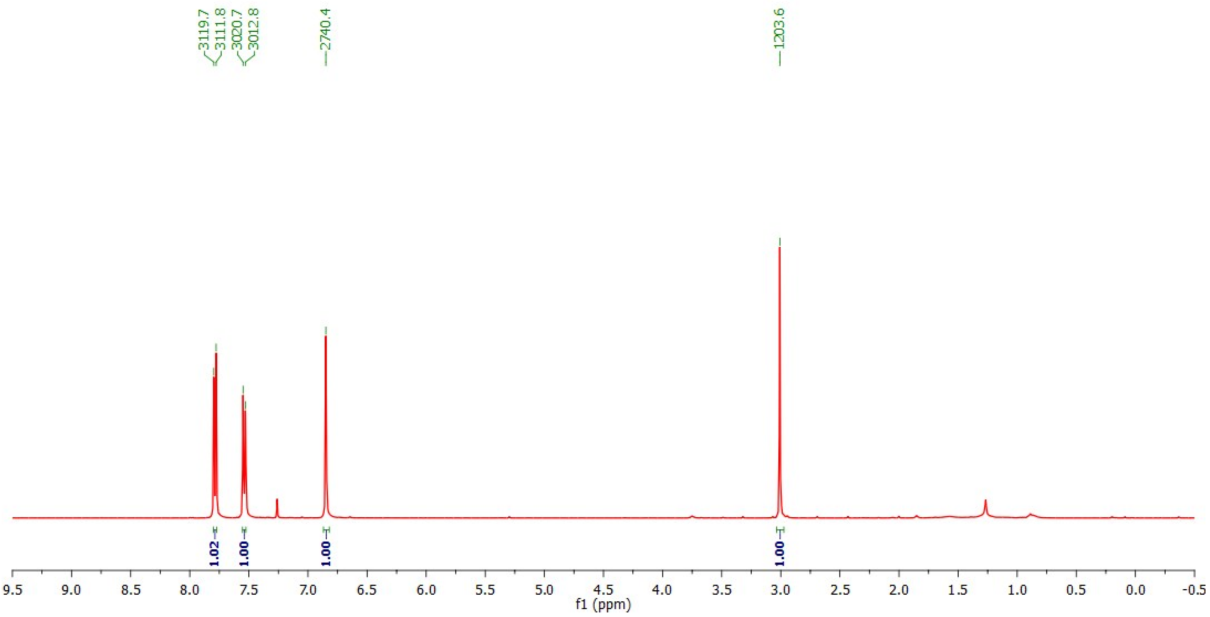

Figure S20.^{1}H-NMR spectrum of compound **3b**.

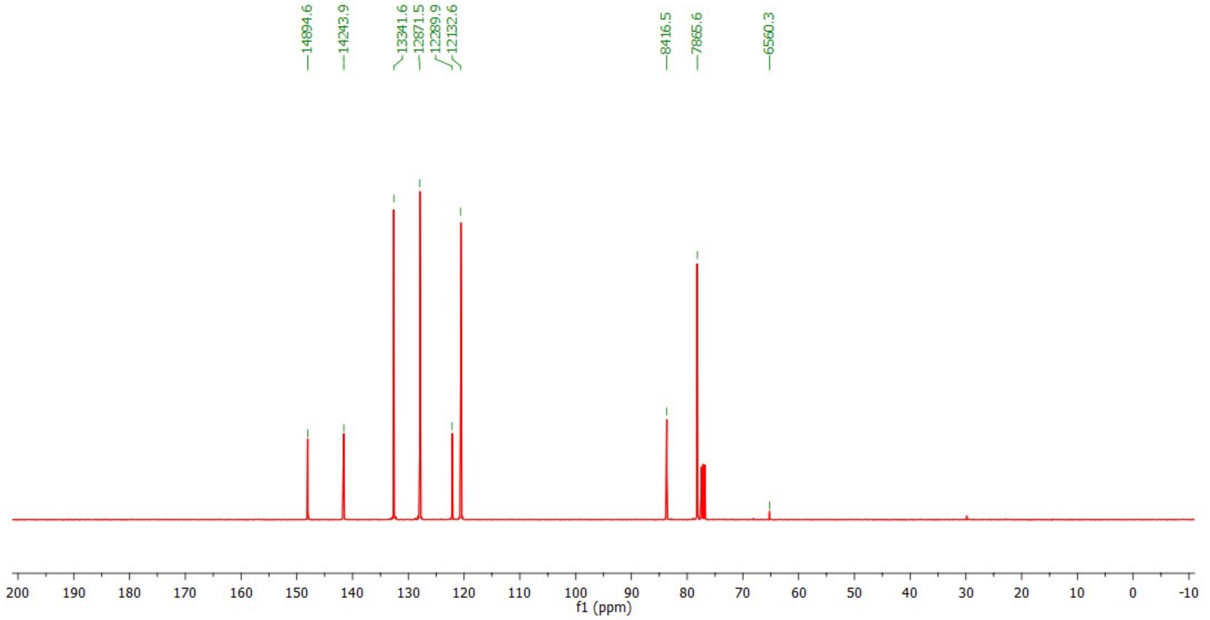

Figure S21.^{13}C-NMR spectrum of compound **3b**.

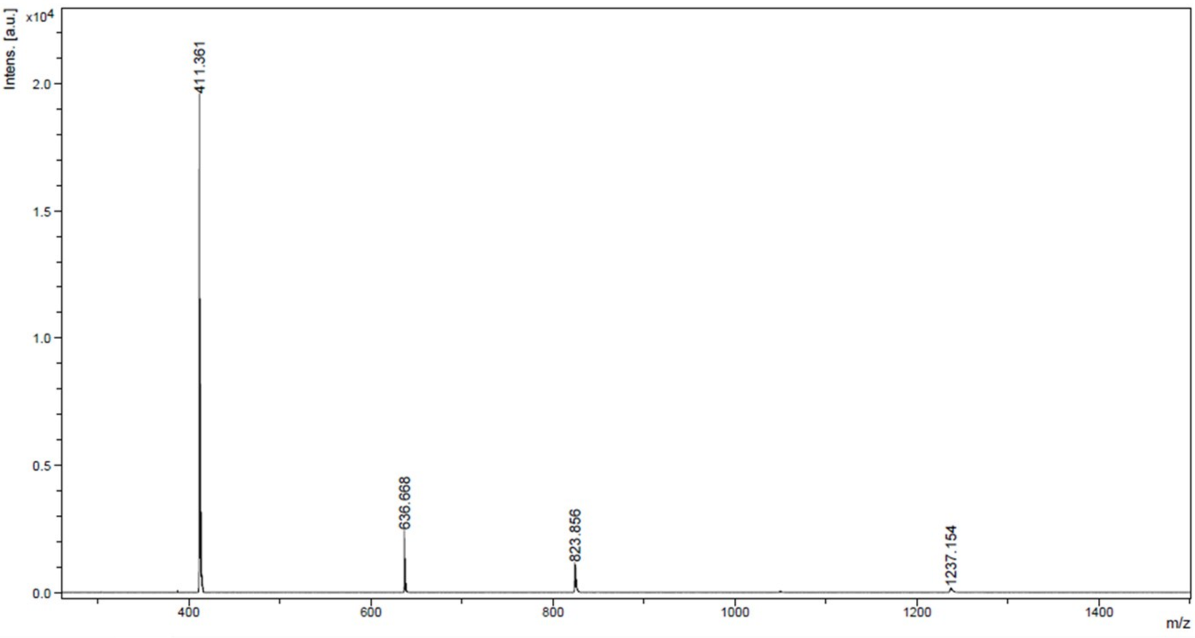

Figure S22. Mass spectrum of compound **3b**.

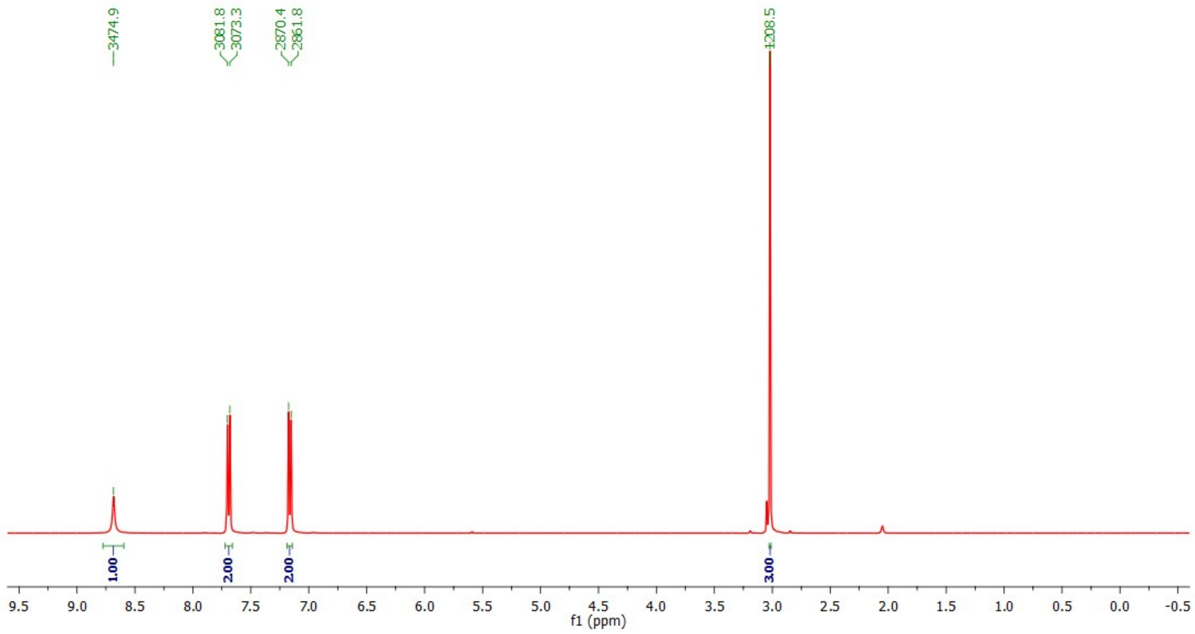

Figure S23.^{1}H-NMR spectrum of compound **CH_3SO_2NH-C_6H_4-I**.

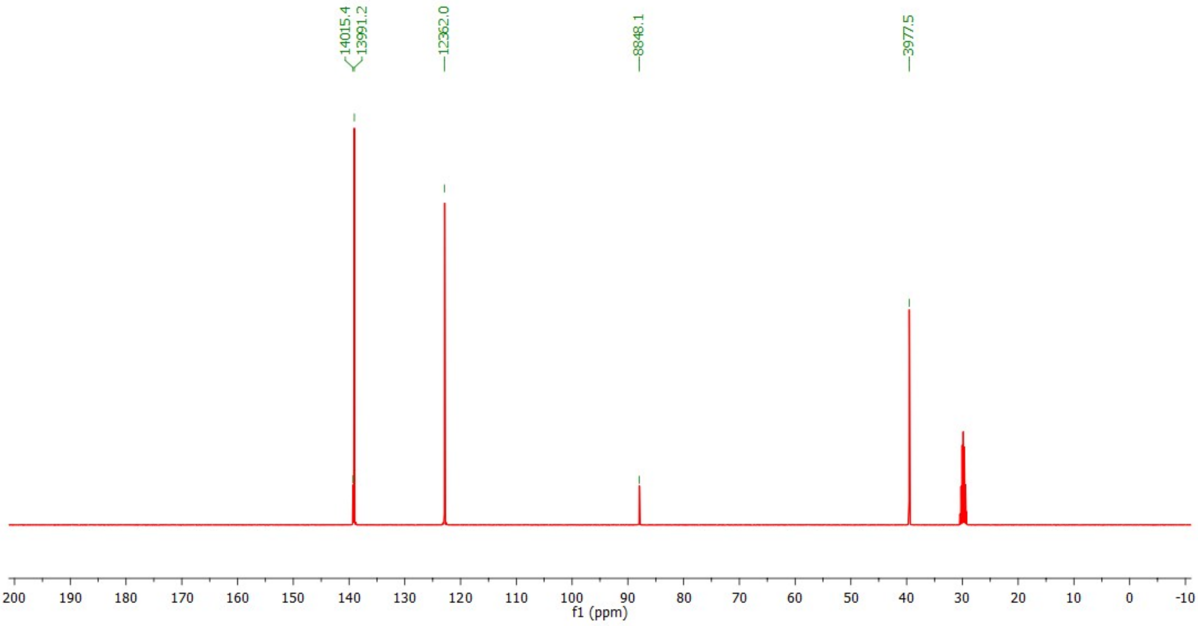

Figure S24.^{13}C-NMR spectrum of compound **CH_3SO_2NH-C_6H_4-I**.

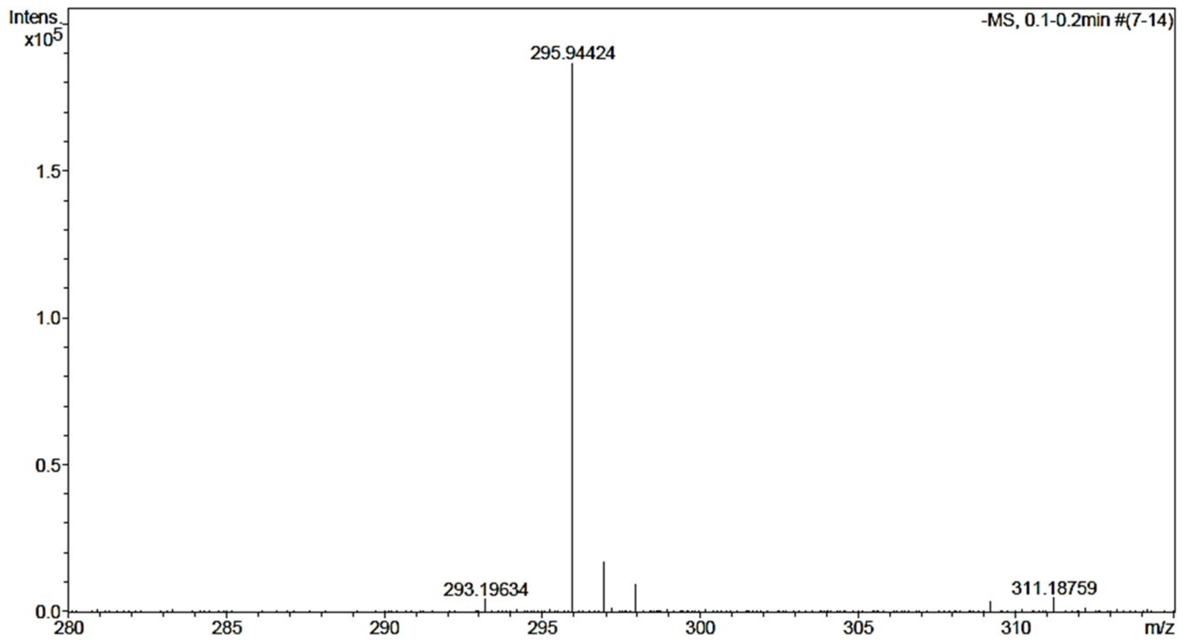

Figure S25. High resolution mass spectrum of compound **CH_3SO_2NH-C_6H_4-I**.

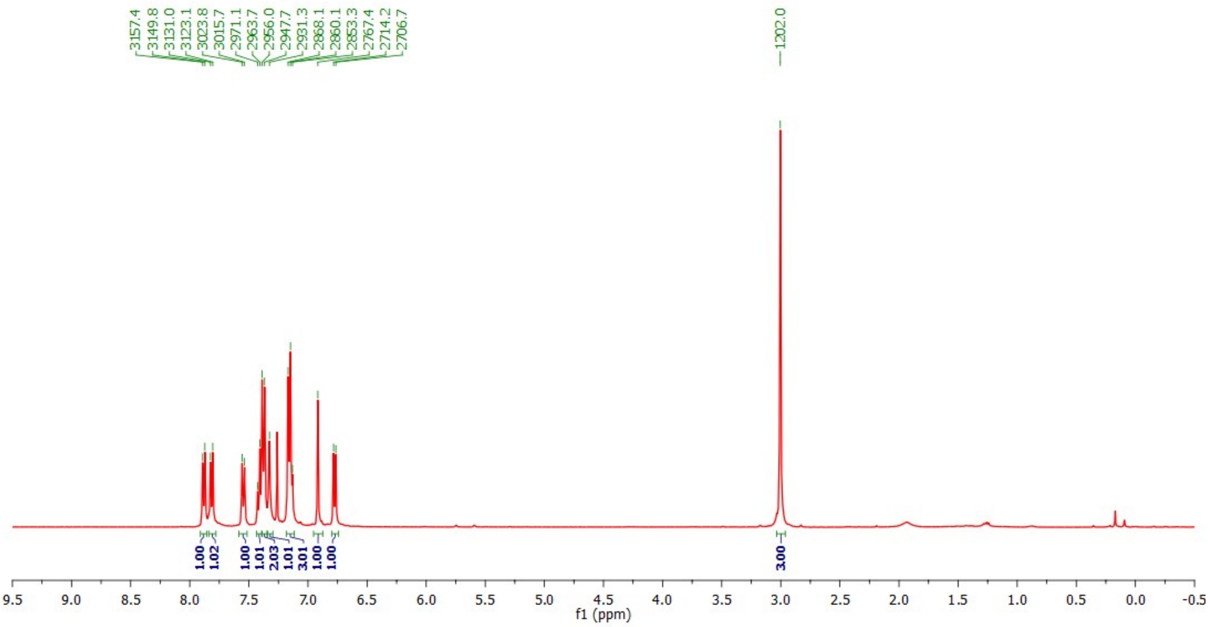

Figure S26.^{1}H-NMR spectrum of **DSS**.

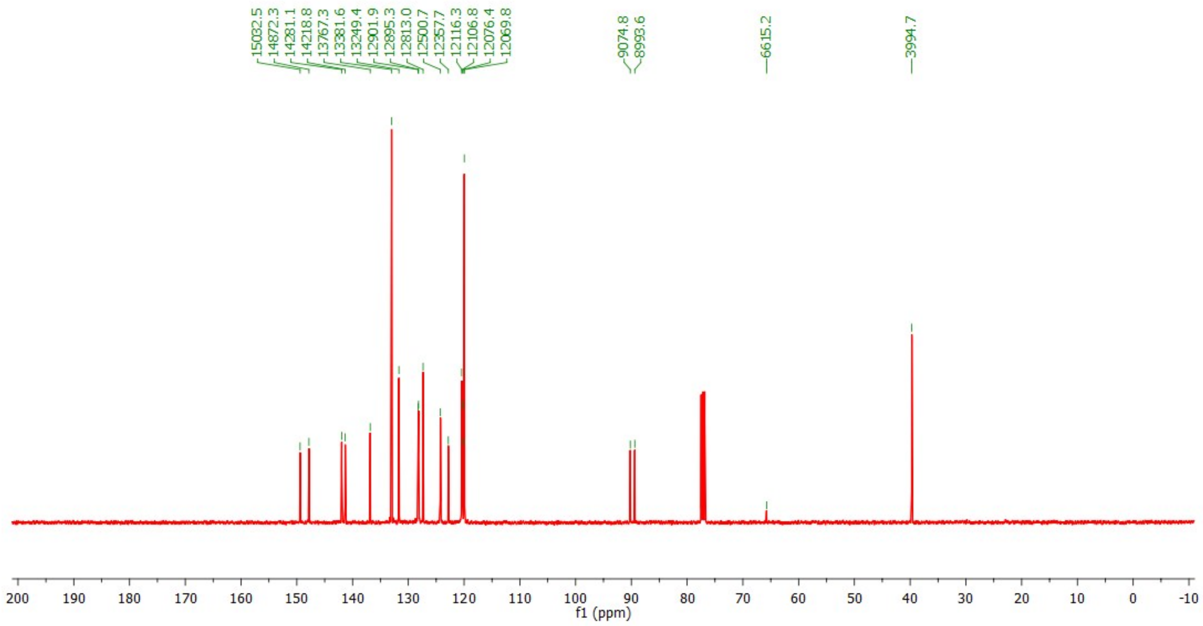

Figure S27.^{13}C-NMR spectrum of **DSS**.

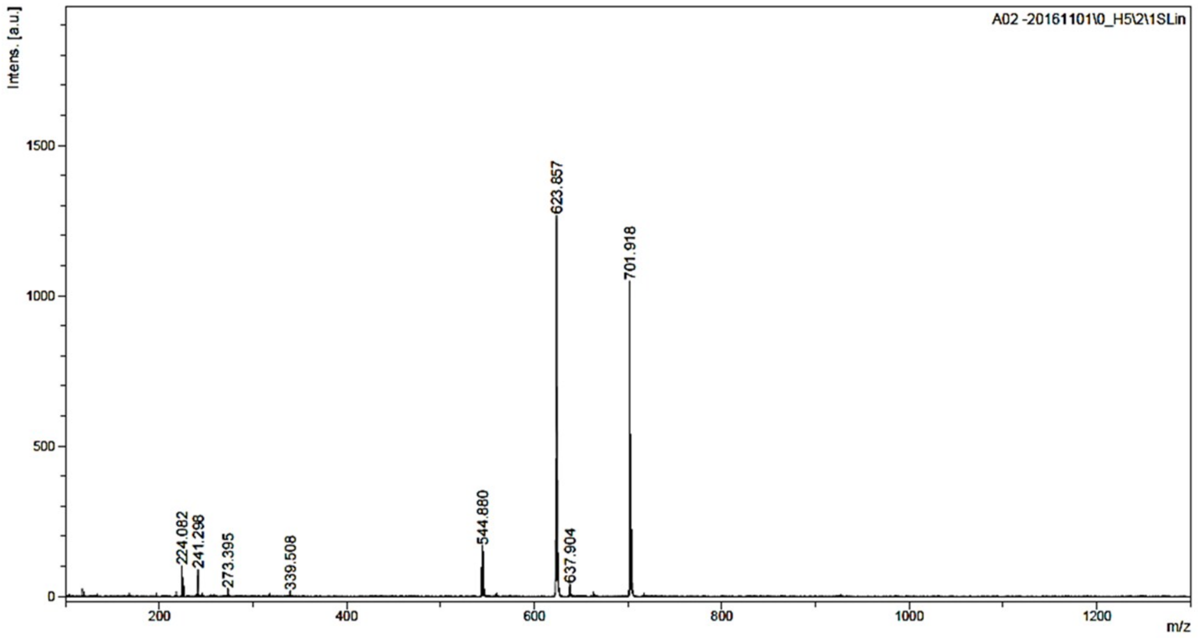

Figure S28. Mass spectrum of **DSS**.

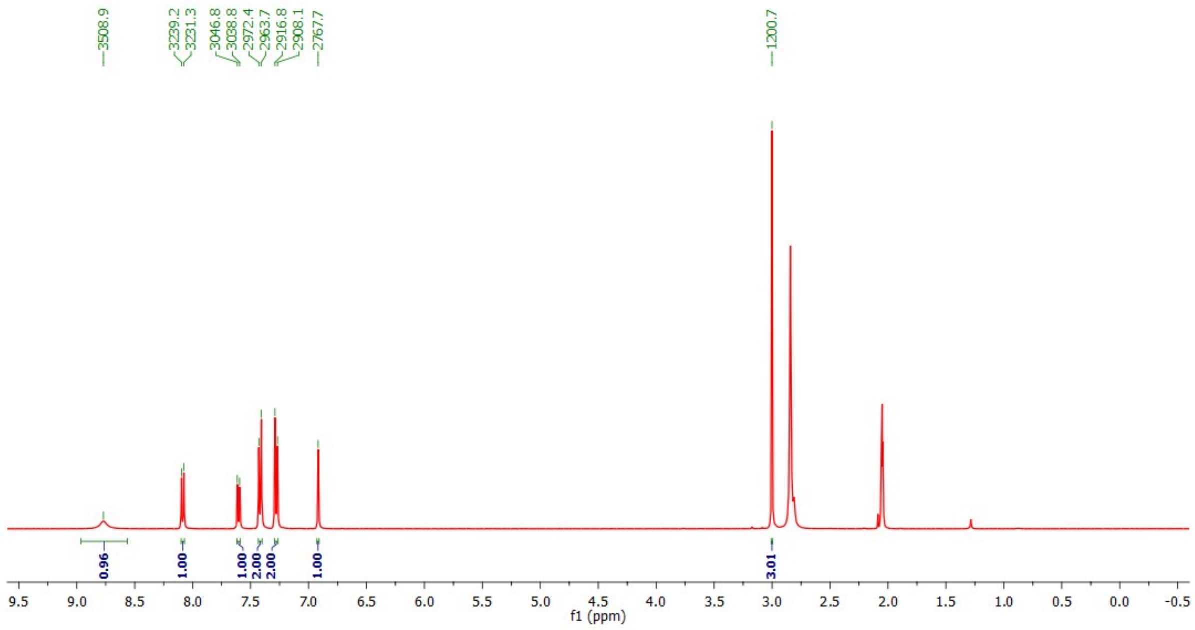

Figure S29.¹H-NMR spectrum of **TSS**.

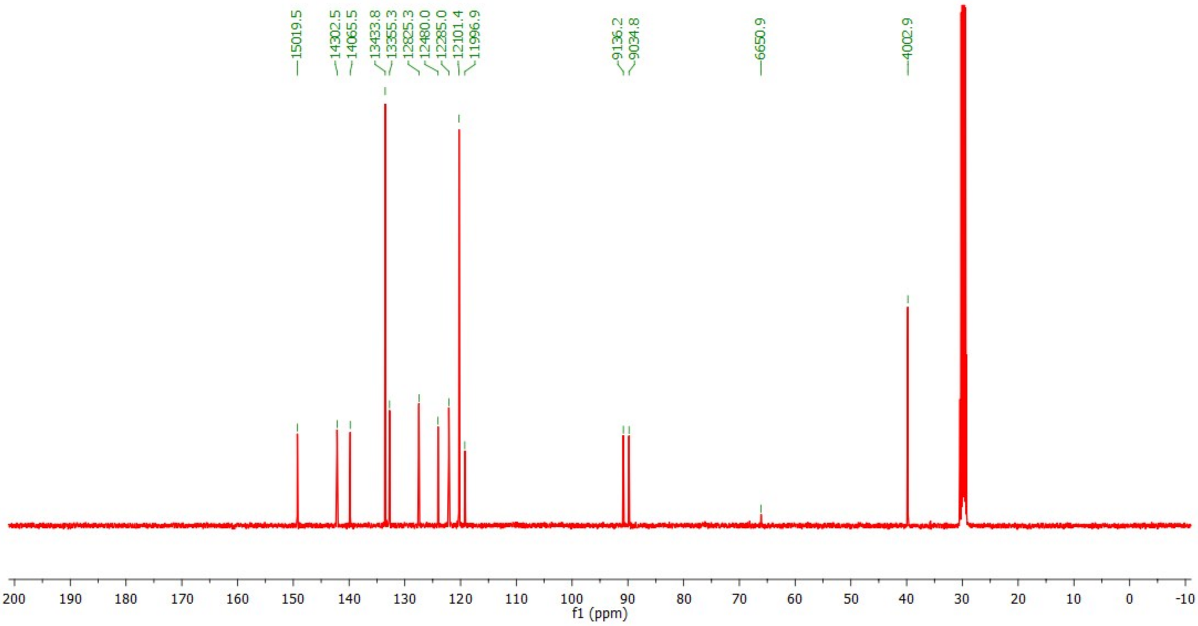

Figure S30.¹³C-NMR spectrum of **TSS**.

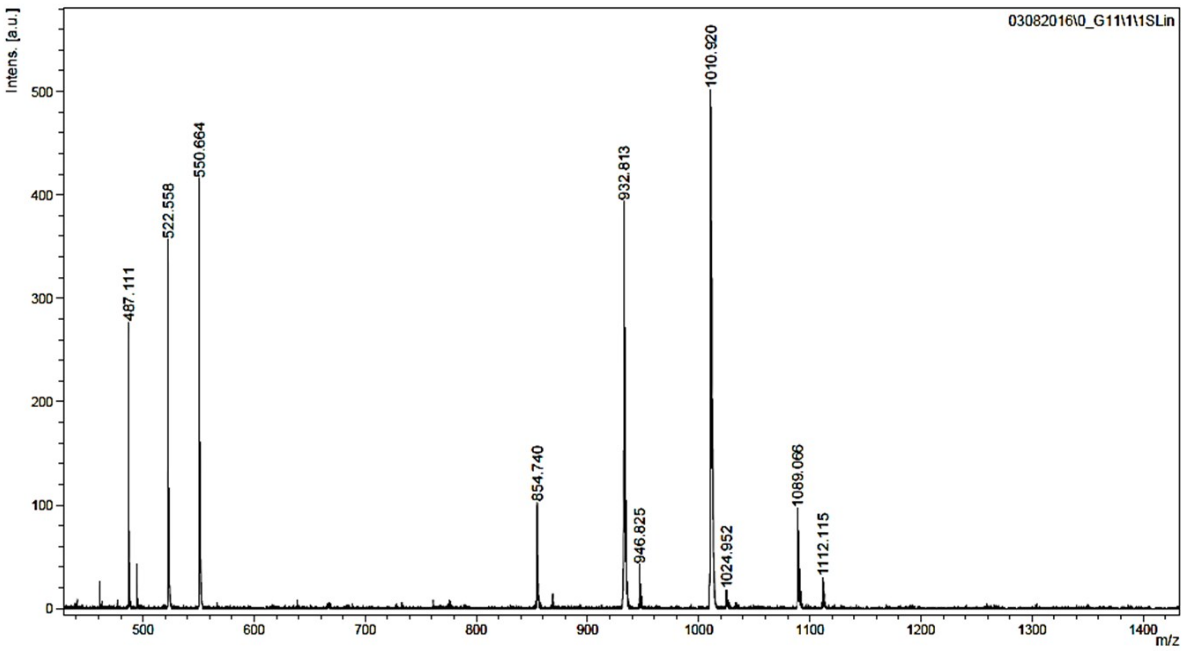

Figure S31. Mass spectrum of **TSS**.
